# Rebalancing the seed proteome following deletion of vicilin-related genes in pea (*Pisum sativum* L.)

**DOI:** 10.1093/jxb/erae518

**Published:** 2024-12-21

**Authors:** Tracey Rayner, Gerhard Saalbach, Martin Vickers, Pirita Paajanen, Carlo Martins, Roland H M Wouters, Catherine Chinoy, Francis Mulholland, Mark Bal, Peter Isaac, Petr Novak, Jiří Macas, Noel Ellis, Burkhard Steuernagel, Claire Domoney

**Affiliations:** Department of Biochemistry and Metabolism, John Innes Centre, Norwich Research Park, Norwich NR4 7UH, UK; Department of Biochemistry and Metabolism, John Innes Centre, Norwich Research Park, Norwich NR4 7UH, UK; Department of Computational and Systems Biology, John Innes Centre, Norwich Research Park, Norwich NR4 7UH, UK; Department of Computational and Systems Biology, John Innes Centre, Norwich Research Park, Norwich NR4 7UH, UK; Department of Biochemistry and Metabolism, John Innes Centre, Norwich Research Park, Norwich NR4 7UH, UK; Department of Computational and Systems Biology, John Innes Centre, Norwich Research Park, Norwich NR4 7UH, UK; Department of Crop Genetics, John Innes Centre, Norwich Research Park, Norwich NR4 7UH, UK; Quadram Institute, Norwich Research Park, Norwich NR4 7UQ, UK; Department of Crop Genetics, John Innes Centre, Norwich Research Park, Norwich NR4 7UH, UK; IDna Genetics Ltd, Centrum, Norwich Research Park, Norwich, NR4 7UG, UK; Biology Centre of the Czech Academy of Sciences, Institute of Plant Molecular Biology, 37005 České Budějovice, Czech Republic; Biology Centre of the Czech Academy of Sciences, Institute of Plant Molecular Biology, 37005 České Budějovice, Czech Republic; Department of Biochemistry and Metabolism, John Innes Centre, Norwich Research Park, Norwich NR4 7UH, UK; Department of Computational and Systems Biology, John Innes Centre, Norwich Research Park, Norwich NR4 7UH, UK; Department of Biochemistry and Metabolism, John Innes Centre, Norwich Research Park, Norwich NR4 7UH, UK; Max Planck Institute for Molecular Plant Physiology, Germany

**Keywords:** Amino acid profile, genetic deletion, mutagenesis, pea genome sequence, pea seed, vicilin

## Abstract

Null mutations for genes encoding a major seed storage protein in pea, vicilin, were sought through screening a fast-neutron mutant population. Deletion mutations at four or five vicilin loci, where all vicilin genes within each locus were deleted, were combined to address the question of how removal or reduction of a major storage protein and potential allergen might impact the final concentration of protein per unit of mature seed weight, seed yield, and viability. While the concentration of seed protein was not reduced in mature seeds of mutant lines, indicative of a re-balancing of the proteome, notable differences were apparent in the metabolite, proteomic, and amino acid profiles of the seeds, as well as in some functional properties. Major effects of the deletions on the proteome were documented. The genomic regions which were deleted were defined by whole-genome sequencing of the parental line, JI2822, and its quintuple vicilin null derivative, providing a comprehensive description of each vicilin locus and its genic arrangement. An annotated reference genome has been generated for JI2822, which will serve as a very valuable resource for the research community and support further study of the associated deletion mutant population.

## Teaser

The genome and proteome of vicilin deletion mutants in pea have been defined. Intercrossed mutants lacking up to 19 vicilin genes show no reduction of protein concentrations in mature seeds.

## Introduction

The seeds of pea (*Pisum sativum* L.) and other legumes are a rich source of protein, starch, fibre, and micronutrients, offering a sustainable source of plant-derived nutrition to support the diets of humans as well as farm animals. As demand for alternatives to animal-based food products grows, the availability of genetic variation for seed constituents is becoming more important, as is the extent to which useful variation can be identified and utilized to meet dietary as well as industry needs. The various macromolecules that are laid down during seed development serve an important function during seed storage and germination. If there is co-adaptation associated with the abundance of different storage compounds, then major changes to one or more of these may have a negative effect on seed development, viability, germination success, and ultimately on survival and fecundity. Nonetheless, there is much natural variation in the types of compounds stored, for example oil- versus starch-storing seeds, as well as in the types of proteins that are stored.

The 11S/12S protein family (classified by protein sedimentation coefficient), for example, is found in some monocots (e.g. rice and oat) and in the eudicots (including legumes and *Brassicaceae*). The 7S storage proteins are common in legumes, being also found in distant taxa such as the monocot *Brachypodium*, as well as rice and oat (Gell *et al.*, 2017), but these are notably absent from the seeds of many *Brassicaceae*, including *Arabidopsis thaliana*, which store oil as a major storage component. In this last case, the proteins synthesized are of two main types: the 11S/12S globulins known as cruciferins, which are salt soluble, and albumins or water-soluble proteins (Shimada *et al.*, 2003; Higashi *et al.*, 2006; Nguyen *et al.*, 2015).

In common with many legumes, pea seeds synthesize 7S globulins, constituting a major fraction of the stored protein, in addition to the 11S/12S globulin and albumin protein classes (Krochko and Bewley, 2000). Here the 7S proteins are known as vicilins, whereas the 11S/12S proteins are legumins, and it is clear that both protein classes have a shared evolutionary history with proteins described as cupins, which include those associated with storage in plants other than angiosperms, such as pteridophytes (Bäumlein *et al.*, 1995; Braun *et al.*, 1996; Shutov *et al.*, 1998, 2003). In pea, the 7S or vicilin proteins are synthesized by multiple genes at each of at least five distinct loci (Domoney and Casey, 1985; Ellis *et al.*, 1986; Domoney *et al.*, 2013). While genes within a locus share a very high degree of sequence identity, the loci themselves are more readily distinguished at the sequence level. The encoded proteins are complex, with some being proteolytically cleaved into as many as three smaller polypeptides, and a subset of these may be further glycosylated (Casey and Domoney, 1999; Freitas *et al.*, 2007).

Vicilins have also been associated with allergenic responses in humans (Lopez-Torrejon *et al.*, 2003), with those from pea identified as a source of potential major allergens. Taylor *et al.* (2021) suggest that pea protein should not be described as hypoallergenic. Pea allergens have been named Pis s 1 and Pis s 2, derived from vicilin and convicilin polypeptides, respectively, based on cross-reactivity of IgE to pea and lentil vicilins in a group of patients (Sanchez-Monge *et al.*, 2004). Vicilins from several legume species, including pea, faba bean (*Vicia faba*), vetchling (*Lathyrus cicero*), and chickpea (*Cicer arietinum*), were reported to be immunogenic in weaned piglets (Seabra *et al.*, 2001; Salgado *et al.*, 2002). It has also been suggested that vicilins form amyloid-like structures in cotyledonary cells, associated with poor digestibility (Antonets *et al.*, 2020).

Vicilins may play a defensive role in seeds (Chung *et al.*, 1997; Marcus *et al.*, 1999; Yamada *et al.*, 1999) and have lectin-like properties (Ribeiro *et al.*, 2014). Vicilin-related proteins from cotton (*Gossypium hirsutum*) seeds inhibited the growth of various filamentous fungi (Chung *et al.*, 1997), while a vicilin-derived peptide from *Macadamia integrifolia* (in the *Proteaceae*, an early diverging lineage of flowering plants (Lamont *et al.*, 2024), inhibited the growth of some plant pathogenic fungi *in vitro* (Marcus *et al.*, 1999). Vicilins from cowpea (*Vigna unguiculata*) seeds were shown to bind to chitinous structures and derived metabolites from the bruchid (*Callosobruchus maculatus*) larval midgut (Sales *et al.*, 1996, 2000; Miranda *et al.*, 2020). From the nutritional viewpoint, a dose-response to pea allergens has been inferred from studies involving consumption of immature versus mature seeds (Taylor *et al.*, 2021), where the former contain much lower levels of protein. Thus, we need to consider whether partial retention of potentially allergenic proteins, rather than their complete removal, offers a compromise route to maintaining plant defence functions.

A suite of related mutants in a common genetic background provides opportunities to dissect the contributions made by individual genetic loci to a range of traits. The generation of mutant lines impacting vicilin seed proteins provides a valuable resource with which questions can be addressed regarding their potentially beneficial function *in planta* and their nutritional impact. Null mutations have been identified in natural populations for some seed proteins, for example pea albumin 2 (Vigeolas *et al.*, 2008; Olías *et al.*, 2023) and the major pea seed trypsin inhibitors (Clemente *et al.*, 2015; Olías *et al.*, 2023), where the numbers of expressed genes are low and at a single locus. In general, the impact of mutations affecting single genes within a multigene locus will be related to gene copy number as well as expression levels for individual functional genes. Where there are multiple multigene loci involved in the expression of a family of proteins, such as vicilins, the effect of mutations in individual functional genes may be expected to be further reduced. A vicilin pseudogene which was detected in some pea genotypes has previously been shown to have little impact on protein composition (Chinoy *et al.*, 2011). Mutations affecting other aspects of seed development have also been shown to impact on the relative amounts of vicilin accumulated in pea seeds. Round-seeded lines of pea contain relatively lower amounts of vicilin than do wrinkled-seeded lines carrying genetic defects in starch metabolism (Casey *et al.*, 1998), while a major role for PsABI5 [involved in abscisic acid (ABA) signalling] in the accumulation of vicilin has been demonstrated through the study of pea *abi5* mutants (Le Signor *et al.*, 2017). Pea *abi5* mutant seeds were also altered in the breakdown of chlorophyll, longevity, and sugar composition (Zinsmeister *et al.*, 2016), and so the effect of these mutations on relative vicilin abundance is likely to be a consequence of changes to carbon metabolism (Le Signor *et al.*, 2017).

In order to investigate the consequences of losing sets of related seed proteins, such as vicilins in pea, we used fast-neutron (FN) mutagenesis, which can create large deletions, as the method of choice. In FN mutants, closely linked genes at individual unlinked loci could be deleted and combined in the progeny of crosses. Here we report the generation of such lines, the definition of the deletions involved, and their consequences when mutations are combined.

## Materials and methods

### Generation of mutant lines lacking vicilin and convicilin

An FN-mutagenized population was generated for the pea accession JI2822 at Oak Ridge National Laboratory, using 20 Gy and 25 Gy doses from the ^252^Cf facility. The value of this resource was demonstrated previously, including through the isolation of two mutant lines in which seed protein gene loci encoding a major vicilin and convicilin storage proteins had been deleted (Domoney *et al.*, 2013). Screening of this population was extended, leading to the identification of a suite of five mutant lines in which individual gene loci, described previously as encoding the major vicilin and convicilin storage proteins, had been deleted. In brief, the population was screened with gene-specific primers, using multiplex PCR and fluorescently labelled primers, and samples with missing amplicons were identified using GeneMapper software, as previously described (Domoney *et al.*, 2013). PCR was used to confirm that amplicons corresponding to additional regions of the gene targets were missing and deletion mutations were confirmed for four classes of vicilin loci and the convicilin locus (*VicA–D*, and convicilin, *Cvc*). The correspondence between *VicA–D* and *Cvc* gene sequences and loci described earlier is given in Table 1. The sequences of all primers used are available in Supplementary Table S1.

**Table 1.** Summary genetic and genomic data for vicilin and convicilin gene classes and their deletions in pea, as determined by sequencing the quintuple null mutant and parental lines

Gene class	NCBI reference	Genetic map location (LG/chromosome)	Chromosomal coordinates of locus (start)	Chromosomal coordinates of locus (end)	Locus size (bp)	Functional gene copy number (predicted non- functional in parentheses)	Chromosomal coordinates of deletion (start)	Chromosomal coordinates of deletion (end)	Deletion size (bp)	Distance deletion start to locus start (bp)	Distance locus end to deletion end (bp)
*VicA*	J01258	LGIIIChr5	594 234 841	594 471 396	236 555	3 (1)	593 569 649	594 585 006	1 015 357	665 192	113 610
*VicB*	X14076	LGIIIChr5	189 161 241	189 345 114	183 873	5	188 955 701	189 348 312	392 611	205 540	3198
*VicC*	X67428	LGVChr3	262 634 484	262 876 982	242 498	8 (3)	261 600 621	263 286 852	1 686 231	1 033 863	409 870
*VicD*	Y11207	LGIIChr6	84 062 923	84 064 861	1938	1	83 953 840	84 113 789	159 949	109 083	48 928
*Cvc*	X06398	LGIIChr6	458 306 531	458 352 969	46 438	2	457 683 765	458 876 284	1 192 519	622 766	523 315
*VicE*	87% identity to X14076; 98% identity to XM051026252	LGIIIChr5	150 208 189	150 210 371	2182	1	_	_	_	_	_
Additional deletions in quintuple mutant	_	LGIChr2	_	_	_	_	4 130 035	4 146 932	16 897	_	_
	_	LGIIIChr5	_	_	_	_	48 203 112	48 280 633	77 521	_	_
	_	LGIIIChr5	_	_	_	_	119 117 091	119 254 821	137 730	_	_

The chromosomal coordinates for each gene class are given, from the start codon of the first gene copy to the stop codon of the last copy. Deletion mutants for the *VicA–D* and convicilin (*Cvc*) gene classes were identified and intercrossed. Chromosomal and deletion coordinates are based on the genome sequence assembly of JI2822. Note that, during the assembly of the genome, an additional vicilin gene was discovered, which lies outside the loci described previously. This has been named *VicE*, a single vicilin gene, a close homologue of that identified in the ZW6 pea accession (Yang *et al.*, 2022) as XM051026252.

The FN mutants used in this study were: FN2076/5_1_1/2, VicA; FN3272/1675_1_1/1, VicB; FN1207/2_1_3/2, VicC; FN3534/2653_1_2/1, VicD; FN3082/905_1_1/2, Cvc.

Individual mutations were combined by intercrossing to generate quadruple and quintuple mutant lines. Assays to determine gene copy number (homozygous wild type, homozygous null, or hemizygous states) were established, using gene-specific primers and gene-specific fluorescently labelled probes to facilitate the combination of mutations. The PCRs used the Absolute Q DNA master mix (Thermofisher) and AgpS2 primer set (Supplementary Table S1) as an internal standard and were carried out using a CFX96 instrument (Biorad). First, all combinations of double mutants were generated by crossing single mutant lines (FN2076/5_1_1/2, VicA; FN3272/1675_1_1/1, VicB; FN1207/2_1_3/2, VicC; FN3534/2653_1_2/1, VicD; FN3082/905_1_1/2, Cvc). F_1_ progeny plants were screened using fluorescently labelled primers and deletions in the heterozygotes deduced from quantitative analysis of amplicon peak heights relative to the total peak height for the sample. F_2_ plants were genotyped using TaqMan assays to determine gene copy number, and double nulls were selected. Then quadruple mutants were generated by crossing selected double mutants (VicAD×VicCB, VicBCvc×VicAC, and VicACvc×VicBC). The derived F_1_ and F_2_ progeny plants were screened using TaqMan assays, and lines that were homozygous null for all four gene targets were generated by selfing. A quintuple null mutant was generated by crossing two quadruple mutants (VicABCD×VicABCCvc). Quintuple mutant lines were identified at F_2_ using TaqMan assays. In some cases, individuals that were hemizygous for individual gene targets were crossed to generate homozygous nulls. The progenitor line JI2822 was used as a control in all experiments.

### Growth of experimental plants

Seed yields from control and combined null mutant lines were determined from plants grown in a randomized block design (10 plants per line), under glasshouse conditions, with supplementary heating and lighting in winter months. For field studies, plants were sown in replicated microplots (1 m^2^) during 1–3 growing seasons (April–July 2018, 2019, and 2021).

For amino acid analysis, the parental control (JI2822) and quintuple mutant lines were sown in triplicate microplots (1 m^2^, 100 seeds) in three seasons (2018, 2019, and 2021) and seeds were harvested at maturity. Seed samples for the quadruple null mutant, VicABCcV, were harvested from microplots sown in 2 years (2019 and 2021), and from an additional quadruple mutant, VicABCD, in 2021.

For gene expression analysis, control and quintuple mutant plants were grown in the glasshouse, as above. Flowers were tagged when they were fully open and pollinated. Pods were collected at 14, 21, 28, and 35 d after flowering. Embryos (cotyledons and embryonic axes) were dissected from developing seeds for RNA preparation, frozen in liquid nitrogen, and stored at –80 °C.

### Analysis of seed proteins

Cotyledonary samples were prepared by drilling meal from five mature seeds per sample, using three biological replicates for every genotype and three technical replicates for every sample. Proteins were extracted and measured, as described previously (Olías *et al.*, 2023). The percentage protein per unit dry weight was calculated for every sample. (For protein determination in bulk samples prior to amino acid analyses, see below.)

One-dimensional denaturing gel analyses of pea proteins were carried out using 12% Bis–Tris pre-cast gels (Invitrogen), according to the manufacturer’s instructions with NuPAGE MOPS (Invitrogen) as running buffer. Immediately before loading, samples were reduced with DTT; NuPAGE antioxidant was added to the upper buffer chamber to prevent re-oxidation of reduced proteins during electrophoresis. Gels were stained using InstantBlue (Abcam, UK). Protein standards used in gel analyses were SeeBlue™ pre-stained standards ranging from 3 kDa to 198 kDa (Thermofisher).

For quantitative analysis of proteins on two-dimensional gels, seed meals (six seed samples, three biological replicates) were extracted in sample buffer [5 mg ml^–1^ 7 M urea, 2 M thiourea, 2% (v/v) CHAPS, 1/200 final volume immobilized pH gradient (IPG) buffer (GE Healthcare) at pH range 3–10, 18.2 mM DTT, 0.01% (w/v) bromophenol blue] for 30 min, at room temperature, with brief mixing. Following centrifugation at 13 400 *g* for 5 min, supernatants were removed and stored at –80 °C. Rehydration of IPG strips was performed overnight at 20 °C, using 125 µl or 200 µl of seed extract supernatant for 7 cm and 11 cm IPG strips, respectively. Strips were incubated under a layer of DryStrip Cover Fluid in trays (GE Healthcare).

Isoelectric focusing was performed using the IPGphor focusing unit (GE Healthcare), according to the manufacturer’s instructions, and the focused strips were frozen and stored at –80 °C in sealed 15 ml tubes. Three strips were prepared for each biological replicate. Thawed, focused IPG strips were rinsed in MilliQ water before shaking in reducing buffer [0.122 M Tris-acetate, 0.5% (w/v) SDS, 6 M urea, 3% (w/v) glycerol, 0.01% (w/v) bromophenol blue, 8 mg ml^–1^ DTT] for 30 min at 20 °C, followed by alkylation buffer [0.122 M Tris-acetate, 0.5% (w/v) SDS, 6 M urea, 3% (w/v) glycerol, 0.01% (w/v) bromophenol blue, 25 mg ml^–1^ iodoacetamide] for 30 min at 20 °C in the dark. IPG strips were applied to NuPAGE Bis–Tris 4–12% gels (Life Technologies) and run in MES buffer plus antioxidant, or to Criterion XT 4-12% gels (BioRad) and run in MES-XT Buffer, according to the manufacturer’s instructions, for 7 cm and 11 cm strips, respectively. Mark12 molecular weight standards (Life Technologies) were used for size calibration. Gels were fixed in 40% (v/v) methanol, 10% (v/v) acetic acid for 2 h at 20 °C, stained overnight in Sypro Ruby Protein stain (Life Technologies) at 20 °C in the dark, and destained in 10% (v/v) methanol, 6% (v/v) acetic acid for 2 h at 20 °C prior to image analysis.

The 2D Sypro Ruby-stained gels were imaged using the BioRad Pharos FX Imager, with a GS800 Calibrated Densitometer, and using Quantity One software. A 532 nm laser, with a 605 nm filter and 50 µm resolution, was selected for the final scan, with a laser energy setting ensuring there were no saturated areas within the image. SameSpots software (Totallab) was used to normalize and quantify all spots above a threshold value, and determine statistically significant differences, using nine gels per sample (three replicates of every biological sample). Significantly different spots were picked for identification using MALDI-TOF (matrix-assisted laser desorption ionization-time of flight), utilizing the ProPic Robot (Genomic Solutions), and transferred to 0.2 ml PCR tube strips.

Gel spots from the picker were washed twice in 200 mM ammonium bicarbonate, 50% (v/v) acetonitrile, followed by two washes in acetonitrile, and air dried. Trypsin [modified porcine trypsin, Trypsin Gold (Promega), 5 µl of 10 ng µl^–1^ 10 mM ammonium bicarbonate] was added to each gel sample; samples were incubated for 3 h at 37 °C. Formic acid (5 µl of 5% v/v) was added to every sample and incubated at room temperature for 10 min. Samples were flash-frozen and used for MALDI-TOF analysis. Briefly, sample aliquots of 1 µl were spotted onto a PAC plate (Prespotted AnchorChip™ MALDI target plate, Bruker Daltonics, Coventry, UK), pre-spotted with α-cyano-4-hydroxycinnamic acid MALDI matrix. After 3–5 min, spots were washed with 10 mM ammonium phosphate, 0.1% trifluoroacetic acid (TFA) according to the manufacturer’s instruction. Samples were analysed with a Bruker Autoflex Speed TOF/TOF using an AutoXecute method optimized for peptide analysis in positive ion mode in the *m/z* range 700–4000. Spectra were summed from ~10×500 laser shots. Data were processed in FlexAnalysis (Bruker) to generate annotated spectra. Calibration was performed for every group of four sample spots using the pre-spotted peptide standards. The generated peak lists were used for database searches (https://www.uniprot.org/; MQcontaminants, https://maxquant.org/, as downloads in 2013/14, using Mascot Server 2.4). Search parameters included the enzyme trypsin with 1 missed cleavage, 100 ppm peptide tolerance, and oxidation (M) as variable and carbamidomethylation (C) as static modifications.

### Proteomic analyses of seed proteins

Proteins were extracted from pea meal, using a phenol-based method (Hurkman and Tanaka, 1986). Briefly, 400 µl of extraction buffer (50 mM HEPES pH 7.5, 5% SDS, 5 mM EDTA, 50 mM mercaptoethanol, Roche protease inhibitor cocktail) was added to 25 mg of pea meal, the suspension was heated at 100 °C for 10 min, and each tube was sonicated with a probe for 1 min. The suspensions were centrifuged, 250 mg sucrose was added and dissolved, followed by addition of 500 µl phenol (Tris-buffer equilibrated, Merck); the samples were vortexed for 15 min, and spun at 10 000 *g* for 10 min. The upper phase (phenol) was carefully removed to a new tube. Four volumes of methanol/0.1 M ammonium acetate were added to the supernatant, and the samples placed at –20 °C for 16 h. The precipitated proteins were collected by centrifugation, washed with methanol and acetone, and dissolved in 200 µl of 20 mM sodium phosphate buffer (pH 8) with 5% sodium deoxycholate. Protein concentrations were determined using the Direct Detect system. Aliquots (100 µg) were reduced with DTT, alkylated with iodoacetamide, and digested with trypsin (Promega). Labelling with iTRAQ (Isobaric Tagging for Relative and Absolute Quantitation, Sciex) or TMT10plex (Tandem Mass Tags, Thermofisher) reagent sets was performed, according to the manufacturers’ instructions, for two independent quantification experiments. Both techniques utilize a multiplexed isobaric chemical tagging reagent, which allows multiplexing of several protein samples for mass spectrometry quantification. In one experiment, four samples were labelled with iTRAQ reagents (two biological replicates each of control and quintuple mutant lines) and, in another experiment, eight samples were labelled with TMT (two biological replicates each of control, two quadruple, and quintuple mutant lines; Supplementary Table S2).

The labelled peptides were desalted using a C18 Sep-Pak cartridge (200 mg, Waters, Wilmslow, UK), and eluted peptides were dissolved in 200 µl of 10 mM NH_4_HCO_3_ and fractionated by high pH reversed phase HPLC. The samples were fractionated on a Waters 626 HPLC system, using an XBridge® 5 µm BEH C18 130 Å column (250×4.6 mm, Waters) and a 60 min gradient of acetonitrile (10–50%) in 10 mM ammonium bicarbonate. Fractions were collected every minute and concatenated to produce 21 (iTRAQ) or 27 (TMT) fractions.

All fractions were analysed by nanoLC-MS/MS on an Orbitrap Fusion™ Tribrid™ mass spectrometer coupled to an UltiMate^®^ 3000 RSLCnano LC system (Thermo Fisher Scientific, Hemel Hempstead, UK). The samples were loaded and trapped using a pre-column with 0.1% TFA at 20 µl min^–1^ for 3 min. The trap column was then switched in-line with the analytical column (nanoEase M/Z column, HSS C18 T3, 100 Å, 1.8 µm; Waters, Wilmslow, UK) for separation using the following gradient of solvents A (water, 0.05% formic acid) and B (80% acetonitrile, 0.05% formic acid) at a flow rate of 0.3 µl min^–1^: 0–3 min 4% B; 3–4 min linear increase B to 7%; 4–80 min increase B to 50%; 80–90 min increase B to 65%; ramp to 99% B; and re-equilibration to 3% B.

Mass spectrometry data were acquired with the following settings in positive ion mode, MS1/OT: resolution 60k, profile mode, mass range *m/z* 400–1800, AGC 4e5, fill time 50 ms; MS2/IT: data-dependent analysis with the following parameters: quadrupole isolation window 1.6 Da, top15 in IT Turbo mode, centroid mode, charge states 2–6, threshold 1.9e4, CID CE=35%, AGC target 1.5e4, max. inject time 105 ms; dynamic exclusion 1 count for 7 s, mass tolerance 7 ppm; MS3 synchronous precursor selection (SPS): 8 SPS precursors, isolation window 1.2 Da, HCD fragmentation with CE=60%, OT 50k profile mode, AGC target 7e4, max inject time 200 ms.

Raw data were processed for protein identification and quantification using Proteome Discoverer 3.1 (Thermo Scientific, PD3.0); all the tools mentioned in the following workflows are nodes of the proprietary Proteome Discoverer (PD) software. The *Pisum sativum* protein database [Proteome ID UP001058974, based on pea cultivar Zhongwan 6 (ZW6; Yang *et al.*, 2022), 64 176 entries] was downloaded from Uniprot.org (August 2023) and used primarily for peptide identification. Proteome ID UP001058974 was imported into PD, adding a reversed sequence database for decoy searches. A database for common contaminants (https://maxquant.org, 245 entries) was also included. For the TMT-labelled samples, the database search was performed using the incorporated search engine CHIMERYS (MSAID, Munich, Germany). The processing workflow included spectrum selection and reporter ion quantification using the most confident centroid (20 ppm). The Top N Peak Filter was used with 15 peaks per 100 Da, and the Inferys_2.1.1_fragmentation prediction model was used with a fragment tolerance of 0.5 Da, using the enzyme trypsin with one missed cleavage, variable modification oxidation (M), and fixed modifications for carbamidomethyl (C) and TMT10plex on the N-terminus and K residues. Identifications were calculated by CHIMERYS for false discovery rate (FDR) 0.01 (strict) and 0.05 (relaxed).

The database search with the iTRAQ-labelled samples was performed using Mascot Server 2.8.0 (in-house). Spectra were pre-processed using the Spectrum RC (recalibration) and Top N Peaks Filter (20/100) nodes. The Mascot search was based on the enzyme trypsin, up to two missed cleavages, fragment tolerance of 6 ppm/0.6 Da, with oxidation (M) and deamidation (N/Q) as variable modifications but fixed for carbamidomethyl (C) and iTRAQ 4plex group. The database search with the TMT samples was performed using the CHIMERYS node (MSAID, Munich, Germany) with the Inferys_2.1_fragmentation model, based on the enzyme trypsin with one missed cleavage, fragment tolerance of 0.6 Da, with oxidation (M) as a variable modification, and carbamidomethyl and TMT10plex (K residue, N-terminus) as fixed modifications. Reporter ions (iTRAQ, TMT) were extracted using the most confident centroid with 20 ppm integration tolerance. Matches were evaluated using Percolator based on *q*-values.

The consensus workflow included the following parameters: assigning two replicate samples per control or mutant line (Supplementary Table S2) and unique peptides (protein groups) for quantification, determination of intensity-based abundance, application of channel correction values (iTRAQ standard, TMT Lot SF239894), establishment of co-isolation/SPS match thresholds 50%/70%, use of normalized CHIMERYS coefficient threshold 0.8, normalization on total peptide abundances, calculation of protein abundance-based ratio, and imputation of missing values by low abundance resampling. For comparisons between mutant and wild-type samples and hypothesis testing, *t*-tests (protein background-based) were used and the adjusted *P*-values were calculated in PD using the Benjamini–Hochberg method.

The results were exported into a Microsoft Excel table, which included data for normalized and un-normalized abundances, ratios for the specified conditions, the corresponding *P*-values and adjusted *P*-values for comparisons of normalized abundances between mutant and control values, number of unique peptides, *q*-values and PEP-values from CHIMERYS, CHIMERYS identification scores, and FDR confidence filtered for high confidence (strict FDR 0.01) only. Further filtering included removal of contaminants and single unique peptide matches.

### Amino acid analysis

Amino acid analyses were performed by Evonik Industries AG (www.evonik.com; https://animal-nutrition.evonik.com/en). Seed samples for control and quintuple mutants were harvested from three separate seasons of field trials and three microplots of each per year. Seed samples for the quadruple null mutant, VicABCcV, were harvested from microplots sown in 2 years, and from an additional quadruple mutant, VicABCD, in 1 year.

Seed samples (100 g) were milled, dried at 103 °C for 4 h, and re-weighed, according to the industry’s standard methods. For these bulk samples, crude protein in seed meals was estimated using the Dumas Combustion Method (European Commission, 2009), based on nitrogen determination.

Amino acid concentrations were determined after oxidation and acid hydrolysis of the samples (AOAC, 1995). Sixteen amino acids were quantified for every sample, whereby asparagine and glutamine were completely converted to aspartic and glutamic acid, respectively, and the latter two values include the former. The quantification of methionine and cysteine is facilitated by oxidation with performic acid prior to hydrolysis, which prevents their degradation, and their derivatives methionine sulfone and cysteic acid are analysed, following protein hydrolysis. As tyrosine is degraded by the oxidation step, and tryptophan is completely destroyed by acid hydrolysis, these two amino acids were not quantified. Amino acids were separated by chromatography on a cation exchange resin column using an Amino Acid Analyser (Biochrom 30) instrument, quantified using integration software, according to the industry’s standard method, and the response factor for each amino acid was calculated.

### DNA extraction and sequencing

A high-quality reference genome sequence was determined for JI2822, using PacBio HiFi and chromosome conformation sequencing (Hi-C). For HiFi sequencing, high molecular weight DNA was prepared from frozen leaves, which were ground in 10 ml of buffer [10 mM trisodium citrate, 5 mM EDTA, 0.03% (w/v) Triton X-100, pH 6.0, containing 0.1% (w/v) BSA and 0.6M mannitol]. The suspension was filtered through Miracloth, centrifuged at 805 *g* for 5 min, and the pellet resuspended in 2 ml of extraction buffer (0.5 M NaCl, 0.1 M Tris–HCl, 0.05 M EDTA, pH 8.0). SDS was added (50 µl of 20% SDS per ml of extract) and mixed before adding 10 µl of DNase-free RNase A (Qiagen 19101) and incubating for 10 min at room temperature. The supernatant was extracted sequentially with phenol, buffered with extraction buffer, and chloroform:isoamyl alcohol (24:1), with gentle inversion and centrifugation at 2300 *g* for 5 min each time, and using wide-bore tips throughout. DNA was recovered from the upper phase with two volumes of ice-cold ethanol by spooling onto a stick with gentle mixing, air-dried, and resuspended in 100 µl of buffer (10 mM Tris, pH 8.0, 0.1 mM EDTA) at 4 °C.

PacBio HiFi genome sequencing was performed (Earlham Institute, Norwich, UK), using the high molecular weight DNA, following a size exclusion step to eliminate DNA fragments <40 kb. Three SMRT cells on PacBio Sequel II were used to generate 96 Gb of data.

For scaffolding of the contigs obtained from the JI2822 HiFi sequencing, DNA was prepared by cross-linking ground leaf tissue according to Miele and Dekker (2009). The stabilized cross-linked DNA was sent to Phase Genomics (https://phasegenomics.com/; Seattle, WA, USA) for Hi-C library preparation. Library preparation followed the Phase Genomics Hi-C (plant) protocol (Van Berkum *et al.*, 2010). Three restriction enzymes (*Dpn*II, *Dde*I, and *Hin*fl) were used to fragment cross-linked DNA. Subsequently, the library was used for Illumina 250 bp paired-end short-read sequencing.

### Genome assembly

PacBio HiFi reads were assembled using HifiAsm version 0.13 (Cheng *et al.*, 2021) and default parameters. Assembled contigs were further scaffolded using the 3D-DNA pipeline (Dudchenko *et al.*, 2017) and Juicebox (https://github.com/aidenlab/Juicebox) for manual curation. Summary data for the resulting high-quality genome are available in Supplementary Fig. S1 and Supplementary Table S3.

Blobtools (version 1.1.1) (Laetsch and Blaxter, 2017) was used to inspect the assembly for contamination. We used short-read data (ERR13156099–ERR13156103) and the NCBI nucleotide collection (downloaded 21 October 2022) as input data. We mapped short-read data to contigs using bwa version 0.7.17 (Li and Durbin, 2009) with default parameters, and sorted SAM files using samtools sort version 1.9. No contamination was found. Scaffolds without hit to any taxa were found to be of low complexity and were removed from the assembly. Scaffolds were also aligned, using blastn, to published pea organelles (MW073102.1 and MW292562.1), and those with >90% of their length matching were removed from the assembly.

The completeness of the assembly and annotation was assessed using Busco version 5.4.7 (Manni *et al.*, 2021) in genome mode, using embryophyta_odb10 database.

### RNA extraction and sequencing

Ten sources of RNA for JI2822 were used, including embryos (cotyledons plus axis) harvested at 14, 21, 28, and 35 d after anthesis from plants grown under glasshouse conditions (as above); 2-week old seedlings, where aerial shoots above soil level were harvested; flowers, harvested at anthesis [defined as full reflex of petals, 24–48 h after cleistogamous self-fertilization, including all floral organs (calyx, corolla, androecium, and gynoecium)]; immature whole pods, which were 20–30 mm in length with style and stigma retained; and embryonic axes which were removed from seedlings, following germination on wet filter paper for 5 d at room temperature in the dark. In addition, nodulated and non-nodulated roots were obtained from sterilized seeds, following germination in the dark for 5 d on filter paper before transferring to sterile flasks containing Fahraeus medium (Young *et al.*, 1982; 100 ml in 250 ml conical flasks with a foam bung) for a further 5 d under dark conditions. Nodulation was induced following addition of *Rhizobium leguminosarum*, strain A3841, which was grown in TY (tryptone-yeast) medium and diluted to an OD_600_ of 0.001 with water, before addition (1 ml) to flasks. For non-nodulated roots, 1 ml of sterile water was added to each flask. Plants were grown under lights for 21 d at 20 °C.

All harvested tissues were flash-frozen and stored at –80 °C prior to being ground in liquid nitrogen. RNA was extracted from seedlings, flowers, pods, and embryonic axes, using a Qiagen RNeasy Plant Mini-Kit, according to the manufacturer’s protocol. RLT buffer was used for extraction of total RNA from seedlings, whereas RLC buffer was used to extract RNA from flowers and pods with a high sugar content. For all other samples, 70–100 mg of ground tissue was extracted using a Spectrum Plant Total RNA kit (Sigma, STRN250), according to the instructions provided (Protocol B, as recommended for starch storage organs).

All RNA preparations were further purified, using an on-column DNase digestion kit (Qiagen kit, 79254) and their concentrations determined using a Qubit and associated RNA BR Assay kit (Invitrogen Q10210). RNA integrity was assessed using RNA TapeStation (Agilent). All samples had a RIN^e^ value >9.5. Individual samples from 10 tissues (4 µg of total RNA) were sequenced using Illumina technology (30 G raw data per sample, Novogene). A pooled RNA sample of nine plant organs (four embryo developmental stages, roots, roots with nodules, seedlings, flowers, and pods) and a single sample from embryonic axes (2 µg of total RNA) were sequenced using Iso-Seq (PacBio; two SMRT cells with 140 Gb per cell, Earlham Institute, Norwich, UK).

### Gene annotation

Gene annotation was performed mainly using Braker3, version 3.0.8 (Gabriel *et al.*, 2024), with default parameters. RNA-seq data from 10 organs, developmental stages, or treatments of pea (as above) were mapped to the JI2822 genome assembly using HISAT2 version 2.1.0 (Kim *et al.*, 2019; Zhang *et al.*, 2021). The resulting BAM files and Uniprot—Viridiplantae (https://www.uniprot.org/taxonomy/33090) were used as evidence of the repeat-masked genome sequence. Repeat-masking was performed according to the methodologies described in Supplementary Protocol S1. Briefly, the most abundant families of tandem repeats were detected with TideHunter (Gao *et al.*, 2019). Full-length copies of long terminal repeat (LTR) retrotransposons were annotated using DANTE_LTR (Novák *et al.*, 2024; Neumann *et al.*, 2019). The complete reference library of repetitive elements (Zenodo repository 10.5281/zenodo.12755082) was used as reference for the RepeatMasker annotation (Smit *et al.*, 2013–2015) of the JI2822 assembly. Final repeat-masking was achieved by merging the annotated regions into a single BED file using the bedtools merge tool (Quinlan and Hall, 2010).

The output from Braker3 was further processed. As the focus of the current study, annotations for vicilin-related genes were manually curated and updated. Vicilin genes were identified using blastn (version 2.2.28) (Zhang *et al.*, 2000). Visual inspection of loci revealed that gene models predicted by Braker3 were spanning highly similar paralogues. We re-mapped RNA-seq data from the embryo 35-day sample, using the HISAT2 parameter, max-intronlen 3000, to avoid false-positive ‘introns’ spanning paralogues. Stringtie, version 2.1.1 (Pertea *et al.*, 2015), was then used to call individual gene models. Gene identifiers were updated to encode species, accession, version of annotation, chromosome, and gene order. Protein sequences were annotated using Eggnogg (http://eggnog-mapper.embl.de/) (Huerta-Cepas *et al.*, 2019; Cantalapiedra *et al.*, 2021). The resulting descriptions were added to a final GFF3 file, with data available in the Zenodo repository (doi: 10.5281/zenodo.12755082).

### Re-sequencing of deletion mutants and deletion calling

To establish the size and nature of the deletions generated in the vicilin and convicilin mutants, sequencing of the quintuple null mutant lacking all five targeted vicilin genomic regions was carried out. Briefly, DNA was prepared from young leaflets, following grinding in liquid nitrogen, and extraction buffer (above) was added. DNase-free RNase was added to the extract, which was incubated at 65 °C for 15 min, and SDS was then added to 1%. An equivalent volume of phenol:chloroform:isoamyl alcohol (25:24:1) was added and mixed. The aqueous phase was separated by centrifugation and DNA was precipitated with ethanol as above. DNA was sequenced using Illumina sequencing technologies (Novogene, Cambridge, UK), aiming for 10× coverage. Deletions were identified by mapping short-read data derived from mutants to the reference assembly, using BWA version 0.7.12 (Li and Durbin, 2009) and default parameters, and SAM files were sorted using SAMtools version 1.9. To identify the deletions, we created 100 kb windows using bedtools version 2.31.0 (Li and Durbin, 2009), and used SAMtools bedcov (Li *et al.*, 2009) to identify the rough locations of the deletions, which were subsequently curated manually to find the exact positions.

### Gene expression analysis

Based on the proteome data, the expression of selected up-regulated genes was investigated, using quantitative reverse transcription–PCR. At each stage of seed development, three biological replicates were used. Embryos were freeze-dried and ground using a pestle and mortar. Approximately 50 mg of each sample was extracted with 700 µl of buffer containing 1 M Tris pH 9.0, 1% SDS, and 10 mM EDTA. After mixing, 350 µl of phenol and 350 µl of chloroform:isoamyl alcohol (24:1) were added and mixed. The aqueous phase was separated by centrifugation at 16 100 *g* for 5 min at 4 °C. RNA was precipitated from the aqueous phase using 50 µl of 3 M sodium acetate and 1 ml of ethanol. The RNA pellet was resuspended in 200 µl of water and then precipitated with 200 µl of 4 M lithium chloride. The RNA pellet was resuspended in 50 µl of water and further purified using an RNeasy mini kit (Qiagen) with DNase I (Qiagen) treatment on the column. RNA was quantified using Nanodrop (ThermoFisher) and cDNA was synthesized from 5 µg of RNA using SuperScript IV reverse transcriptase (ThermoFisher) and an ascorbate peroxidase-based poly(A) tail primer (APX1, Supplementary Table S1). cDNA was diluted 1:25 with water prior to qPCR analysis.

Relative expression analysis of selected classes of gene was carried out using elongation factor (EF1α) and actin as reference transcripts and at least three technical replicates of every biological replicate. Primer sequences for target (QLegJ_2f/2r; PA1qF1/R1) and reference transcripts are available in Supplementary Table S1. SYBR Green JumpStart Taq ready mix (SigmaAldrich) and a CFX96 (Biorad) PCR instrument were used for amplification, and CFX software for analysis. Data were analysed as in Livak and Schmittgen (2001).

### Quantification of carbohydrates

Total starch in cotyledons was measured using a Megazyme kit as described previously (Moreau *et al.*, 2022). Cotyledonary sugars were quantified using LC-MS as described previously (Rayner *et al.*, 2017). Analyses were performed using a minimum of three biological and three technical replicates for every genotype analysed.

### Relative viscosity analysis

Flour was prepared using a Cyclone mill (Retch Twister) and sieved through a 125 µM mesh, using two biological replicates and two technical replicates for quintuple mutant and control samples. Water (25 g) was accurately weighed into a metal canister and 4 g of flour added, briefly mixed with a paddle, and inserted into the rapid visco analyser (RVA Techmaster, Perten Instruments) using standard 1 program (heating to 50 °C and holding for 1 min; heating to 95 °C over 3 min 42 s; holding at 95 °C for 2 min 30 s; cooling to 50 °C over 3 min 48 s; holding at 50 °C for 2 min) with viscosity readings every 4 s.

## Results

### Protein profile of lines with single vicilin or convicilin locus deletions

We previously described the identification of FN-derived deletion mutants in pea, which included deletions of the vicilin *VicB*, *VicD* (also known as CD72/P54), and *Cvc* loci (Domoney *et al.*, 2013). Using the same resources and methodologies, we then identified deletion mutants for the *VicA* and *VicC* loci. Table 1 summarizes the linkage group/chromosomal loci for this gene family, including the coordinates of the deletions at every locus, which were derived from comparisons of the genomic sequences of mutant and parental lines (see later).

**Fig. 1.**
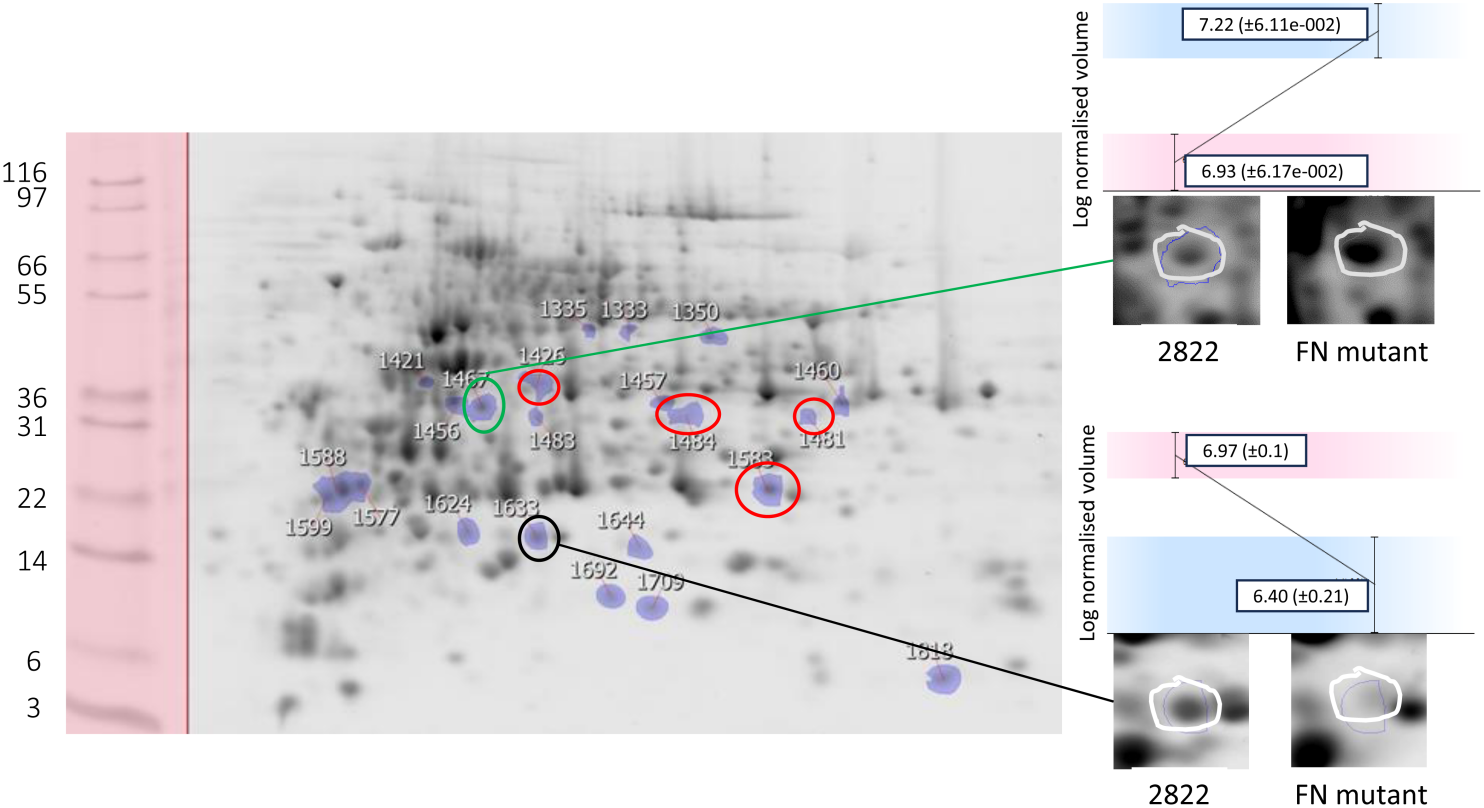
Samespots alignment of a 2D analysis of seed proteins from a mutant identified as lacking *VicA* genes (FN2076/5_1) and JI2822. All significant differences are coloured (purple). Examples of proteins showing significant differences in abundance are highlighted by circles (VicA, black, lower abundance; VicB, red and Leg A, green, higher abundance). Details of two regions highlighted are shown, along with their quantification data (log-normalized spot volume), for one example each of lower and higher abundance proteins (JI2822, left; FN mutant FN2076/5_1, right). Molecular weight markers are shown with sizes indicated on the left (×10^–3^).

Initial evaluation of lines carrying single locus mutations (vicilin or convicilin) was carried out using measurements of total seed protein and comparisons of their constituent proteins by quantitative two-dimensional gel electrophoresis (Fig. 1; Supplementary Figs S2–S5). Analysis of protein concentrations in mature seeds indicated that there was no reduction in total seed protein per unit dry weight in mutant lines compared with the parental line, JI2822 (Supplementary Figs S2A, S5A); in the case of mutants lacking *VicA* and *VicC* genes, a significantly higher concentration of protein (% dry weight) was evident in mutant lines (Supplementary Fig. S2A).

The quantitative two-dimensional gel protein analyses highlighted proteins which were significantly changed in abundance (of either lower or higher abundance), based on overlays of replicate gels of each mutant analysed and control parental samples. Proteins identified as being significantly altered in abundance were selected for identification from control gels. As expected, this analysis showed that proteins which were significantly reduced in abundance corresponded to the gene locus which had been deleted in every case (Fig. 1; Supplementary Figs S2–S4; Supplementary Table S4). Note that, in such quantitative analysis of spot intensity, zero values are rarely achieved (even for null mutants expected to show zero values for corresponding proteins), due to background effects of protein migration, gel staining, and a low level of cross-contamination. These analyses were further complicated by the multitude of vicilin-related protein spots which were significantly reduced in abundance in the case of most mutants, reflecting the extensive proteolytic processing and glycosylation which occurs during the biosynthesis of vicilin (Casey and Domoney, 1999). Besides the expected proteins of reduced abundance, others were found to be present in significantly higher amounts for each of the vicilin deletion classes. For example, for the mutant lacking *VicA* genes, where ~20 proteins were identified as having altered abundance, six showed a higher abundance in the mutant. These were identified as VicB (1), VicC (3), and legumin-related (2) proteins (regions highlighted in Fig. 1), whereas the other differences highlighted by the Samespots analysis mainly reflected VicA proteins of reduced abundance in the mutant, compared with the parental line, JI2822. Summary data for the gel analyses of single mutants are available in Supplementary Table S4.

Analysis of seeds of the mutant lacking *VicB* genes by two-dimensional gel analysis suggested a similar pattern of changes to proteins, with some present in higher abundance, in addition to the expected VicB proteins which were significantly reduced, compared with the parental line. One protein of significantly higher abundance (VicA) and a protein of significantly lower abundance (VicB) in the mutant are highlighted in Supplementary Fig. S2. Ten of 12 proteins of significantly higher abundance were VicA or VicC, one was not identified, and one was a likely contaminant (Supplementary Table S4).

The same analyses of seeds from the mutant lacking *VicC* genes highlighted many differences (41) between mutant and parental lines; of 37 which were investigated, 22 were proteins of lower abundance in the mutant, the majority (18) of which corresponded to VicC. Among the 15 higher abundance proteins identified in the mutant line, legumin, VicA/VicB, and Cvc featured among other unrelated proteins (Supplementary Fig. S3; Supplementary Table S4).

For the mutant lacking the *VicD* gene, the quantitative analysis revealed comparatively few differences from JI2822. Four proteins were identified as being of lower abundance in the mutant, three of which were VicD, along with an elongation factor. VicC was identified as being of higher abundance in the mutant (Supplementary Fig. S4; Supplementary Table S4).

Comparison of the mutant lacking *Cvc* genes with JI2822 revealed many significant differences (49), the majority of which (39) reflected a reduced abundance of convicilin. Three proteins were identified as being of higher abundance in the mutant, two of which were VicA, in addition to VicC (Supplementary Fig. S5; Supplementary Table S4).

Given the complex picture of changes in relative abundance of individual proteins inferred from analyses of the single mutants, where up-regulated proteins included members of vicilin classes other than that directly impacted by the mutation, combinations of mutations were generated to establish the consequences of a near-complete abolition of vicilin synthesis in pea seeds. Combinations of four vicilin/convicilin mutations were generated (Quad ABCD and Quad ABCCvC: lacking vicilins A–D or vicilins A–C plus convicilin, respectively), as well as a quintuple null (Quin) lacking all five genetic loci.

### Protein profile of lines with multiple vicilin/convicilin locus deletions

**Fig. 2.**
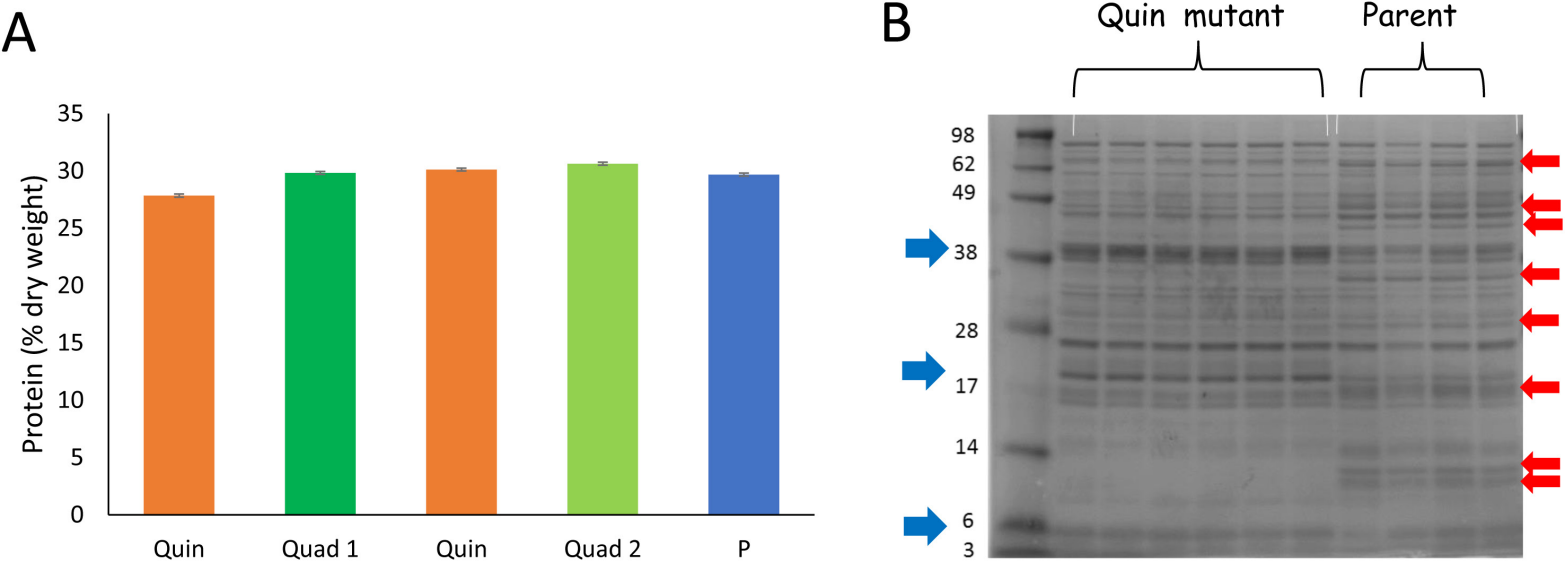
(A) Protein concentration in mature seeds (% dry weight) of Quad 1, lacking vicilins A, B, C, and D; Quad 2, lacking vicilins A, B, C, and convicilin; and Quin mutants, lacking all five vicilins, compared with the parental line, JI2822 (P). Quintuple mutant seeds derived from two independent crosses were analysed as separate replicates; bars, SEM, n. s. (one-way ANOVA). (B) Seed protein profiles of quintuple mutant samples, compared with JI2822. Red arrows highlight proteins missing from mutant samples; blue arrows highlight those additional or higher abundance proteins in mutant samples. Protein standards are shown in the left-hand track with sizes indicated (×10^–3^). Seeds were harvested from plants grown in the greenhouse in the same time interval.

The mean concentration of protein per unit dry weight in mature seeds of the two classes of quadruple (lacking *VicA*, *VicB*, *VicC*, and *VicD* genes or lacking *VicA*, *VicB*, *VicC*, and *Cvc* genes) and quintuple (lacking *VicA*, *VicB*, *VicC*, *VicD*, and *Cvc* genes) null mutants showed no significant difference from each other or from the parental line, JI2822, when plants were grown under greenhouse conditions; values of 28–30% protein (per unit dry weight in mature seeds) were observed in all lines (Fig. 2A). The seed protein profile of the mutants contrasted sharply with that observed for JI2822, with many proteins apparently absent from the mutants, while the mutant samples showed additional, or higher concentrations of other proteins. The seed protein profile of mutants lacking all five loci in comparison with JI2822 is shown in Fig. 2B. The combined data suggested that changes to protein synthesis had occurred in the mutants such that a final seed protein concentration equivalent to that of the parental line was achieved.

**Fig. 3.**
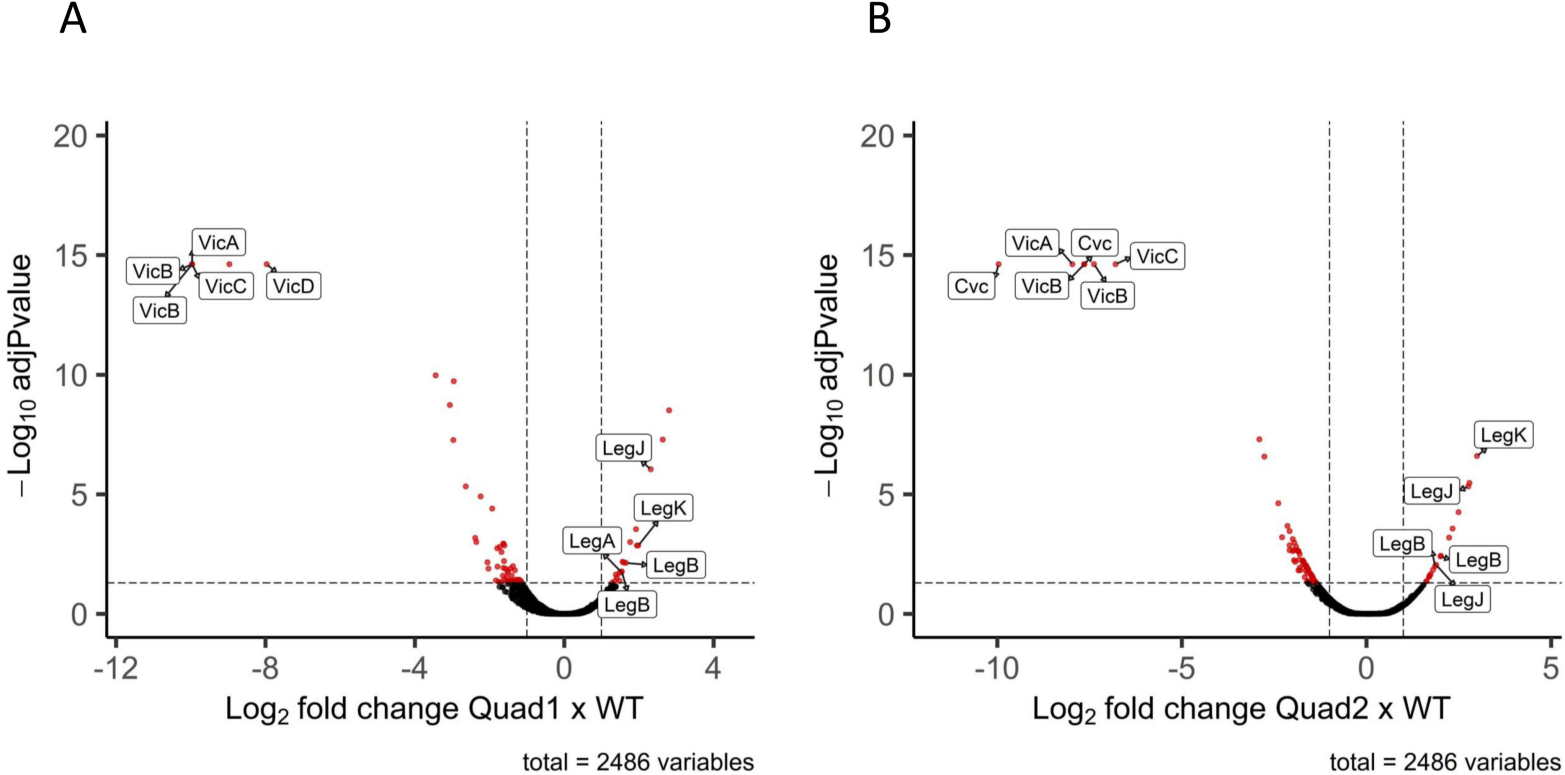
Volcano plots showing the distribution of significant changes to the proteome of quadruple pea mutants compared with parental control lines. (A) Quad 1, lacking vicilins A, B, C. and D. (B) Quad 2, lacking vicilins A, B, C, and convicilin. Vicilin- and legumin-related proteins are labelled.

**Fig. 4.**
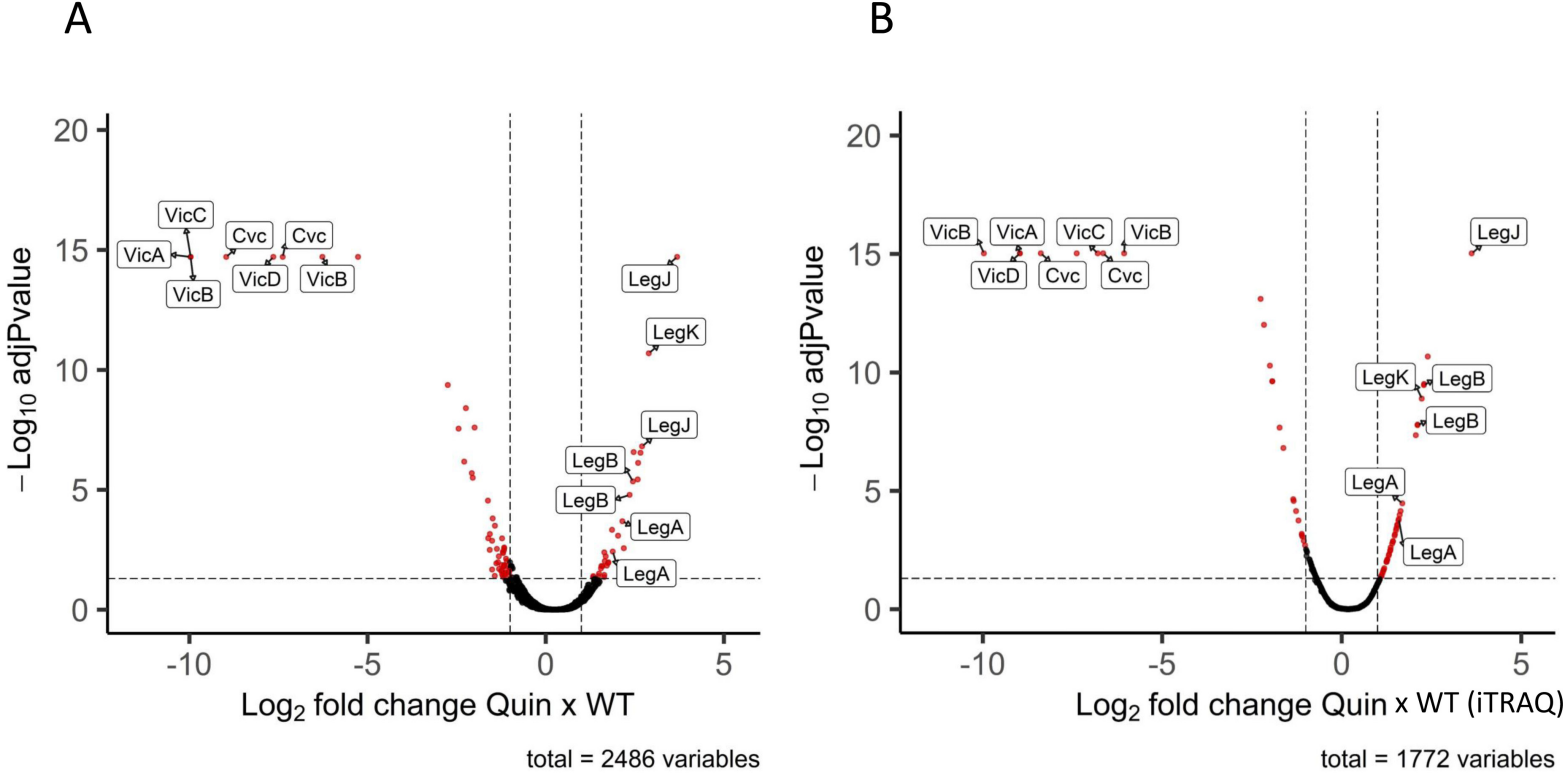
Volcano plots showing the distribution of significant changes to the proteome of quintuple pea mutant compared with parental control lines. Quin, lacking vicilins A, B, C, D, and convicilin. (A) TMT analysis; (B) iTRAQ analysis. Vicilin- and legumin-related proteins are labelled.

In order to identify and quantify the changes, iTRAQ (Quin, and JI2822) and TMT (Quad 1, Quad 2, Quin, and JI2822) labelling of protein-derived peptides was carried out and significant quantitative changes in relative abundance determined. The significantly reduced abundance of the four relevant classes of vicilin proteins, as evident in the case of Quad 1 (lacking VicA, VicB, VicC, and VicD) and Quad 2 (lacking VicA, VicB, VicC, and Cvc), is highlighted in Fig. 3. In these analyses, all proteins which are present in at least one of the two compared samples are shown in the plots, based on the ratios of the abundance of every protein versus the *P*-value determined for their relative abundance in every case. Although zero values are expected for proteins corresponding to deleted genes in the mutants, zero values rarely occur in isobaric labelling due to background noise and channel leakage, and real zero values are imputed with random low values, generating ratios lower than infinite. Additionally, the ratios are capped to a maximum of 1:1000 by the software. In the case of the two quadruple nulls, besides the expected vicilins, additional proteins were present in significantly lower abundance in the mutants, while a lower number of proteins was evidently present in higher abundance (Fig. 3). Of the latter group, legumin proteins are notable in both Quad 1 and Quad 2, with LegJ and LegK at the upper end of the higher abundance proteins. In both labelling analyses of the quintuple nulls, the highly significant reduced abundance of five classes of protein (VicA, VicB, VicC, VicD, and Cvc) was evident (Fig. 4A, B). Again, several legumins were among the proteins of significantly higher abundance, with LegJ and LegK at the upper end of the plots. Together, these data validated the identities of the mutant combinations, with major changes to the vicilin protein classes corresponding to deleted loci in every case.

The identities of the proteins which showed significant differences in the mutant compared with parental lines, together with their fold differences, are given in Table 2 (Quad 1), Table 3 (Quad 2), Table 4 (Quin, TMT), and Table 5 (Quin, iTRAQ) (*P*<0.05). For proteins (18) present in higher abundance in the Quad 1 mutant, the highest fold change was observed for two proteins containing a high proportion of basic and acidic residues: a PXR1-like protein, hypothesized to be involved in peroxisome biogenesis and in directing proteins to the peroxisome (Dodt *et al.*, 1995), and an uncharacterized protein, followed by LegJ and LegK (Table 2). The same pattern was observed for higher abundance proteins (17) in Quad 2, except that the order differed: LegJ and LegK showed a higher fold change than PXR1-like and the uncharacterized protein (Table 3), and this was true also for the Quin mutant (Table 4). The uncharacterized protein is highly repetitive. The structure prediction tool AlphaFold2 confidently predicts a single helix towards the end of the sequence, which shows ~80% identity to a DNA ligase isoform from a number of plants, including *Spatholobus suberectus* (a medicinal plant); this protein and that of pea show a similar abundance of charged residues (K 29/33%, E 17/22%, respectively, and D 12%). Notably, two additional groups of proteins (BURP domain-containing protein and enoyl reductase domain-containing protein) were consistently identified among the TMT-labelled peptides of higher abundance in quadruple and quintuple mutants.

**Table 2.**
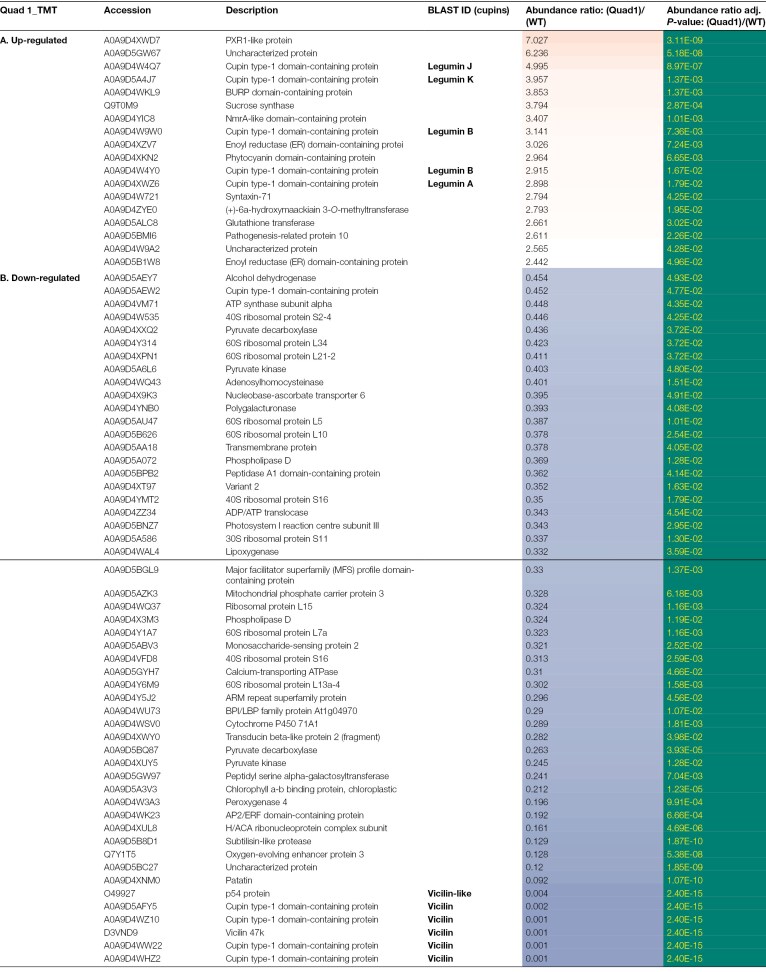
Identities of proteins which show significant differences in abundance in the Quad 1 null vicilin mutant (lacking VicA, VicB, VicC, and VicD) compared with the parental control

**Table 3.**
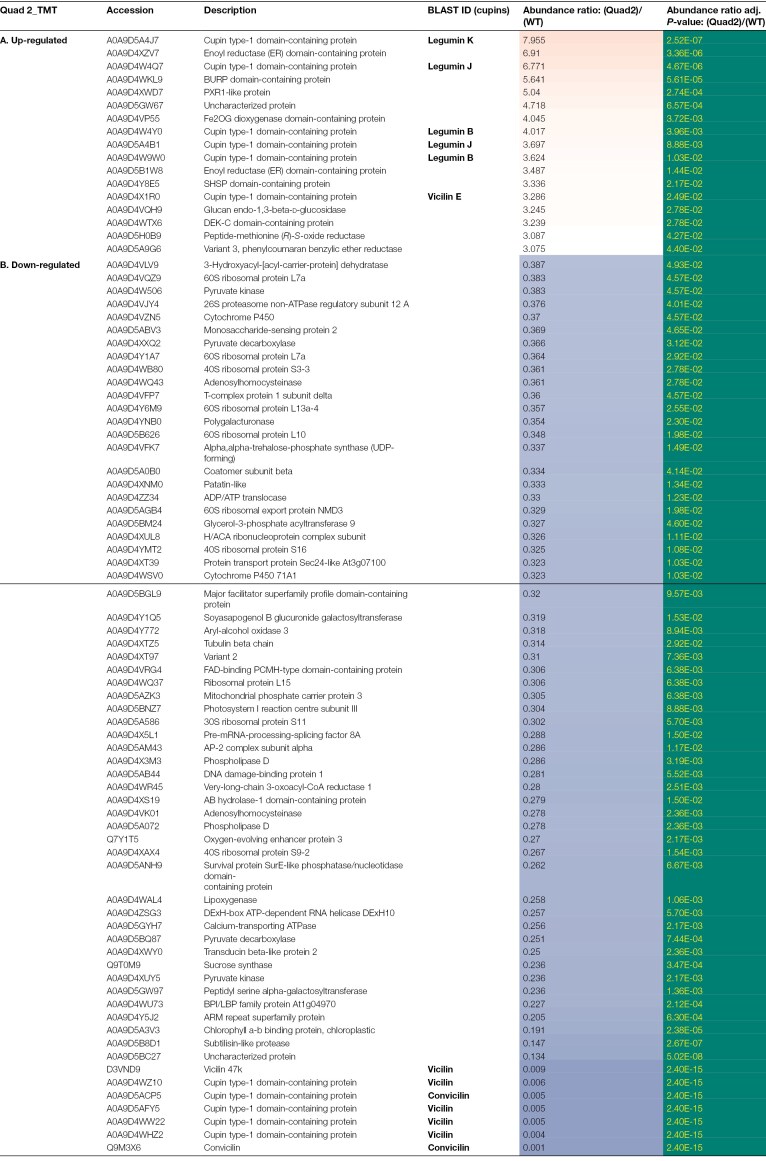
Identities of proteins which show significant differences in abundance in the Quad 2 null vicilin mutant (lacking VicA, VicB, VicC, and Cvc) compared with the parental control

**Table 4.**
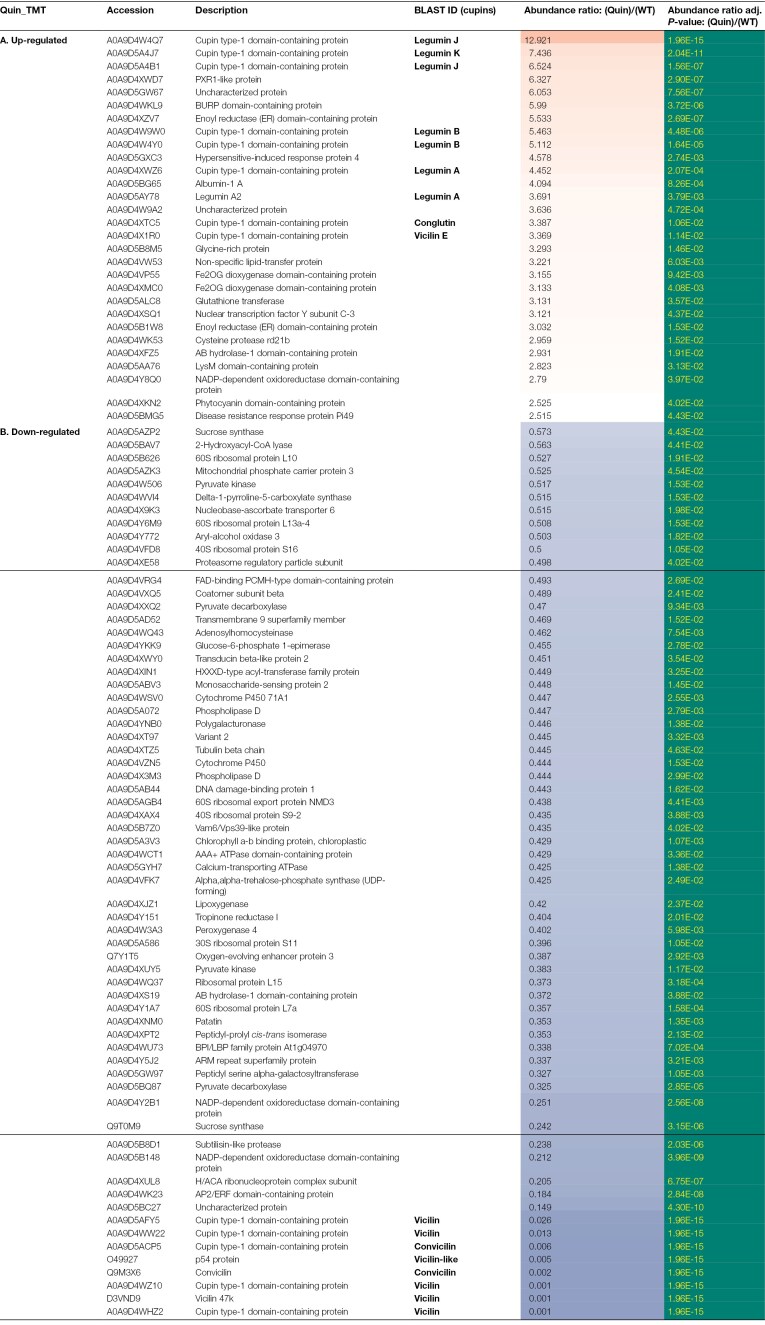
Identities of proteins which show significant differences in abundance in the Quin null vicilin mutants (lacking VicA, VicB, VicC, VicD, and Cvc) compared with the parental control, as assessed by TMT labelling

**Table 5.**
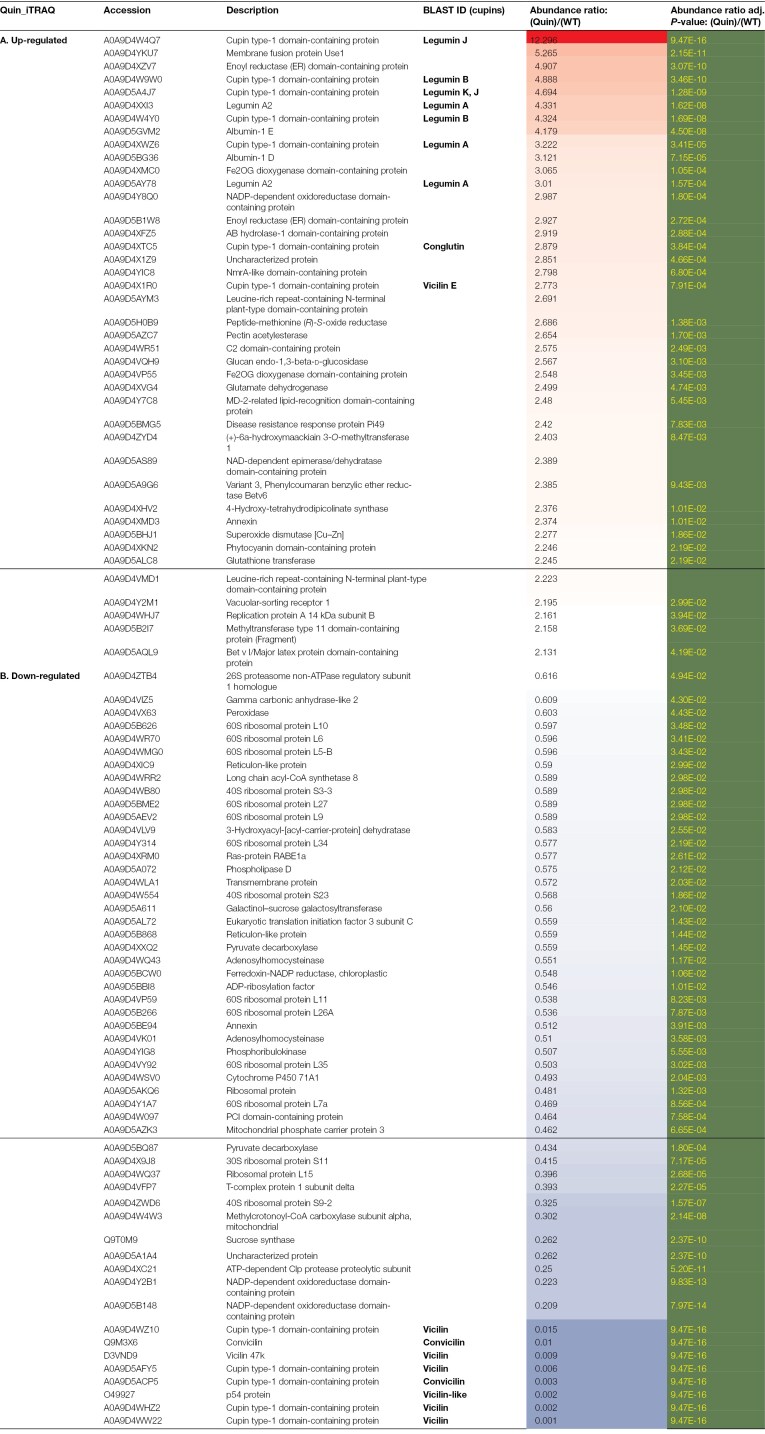
Identities of proteins which show significant differences in abundance in the Quin null vicilin mutants (lacking VicA, VicB, VicC, VicD, and Cvc) compared with the parental control, as assessed by iTRAQ labelling

Analysis of the Quin mutant using the iTRAQ methodology showed a greater number of proteins in the relatively higher abundance class (41, compared with 29 for TMT labelling) but confirmed legumin proteins as those showing the biggest fold change in this group (Table 5). Unexpectedly, analysis of Quad 2 and Quin mutants revealed a hitherto unidentified vicilin protein among the significantly higher abundance proteins, and this was true for both analyses of the Quin mutant, with an ~3-fold increase in abundance in all cases (Tables 3–5). This vicilin, which did not feature among significantly affected proteins in the Quad 1 mutant, has been named VicE, and is encoded by a single gene at a locus on chromosome 5 (distinct from the loci encoding VicA or VicB) in the genome sequence of JI2822 (Table 1).

Of the proteins which showed highly significantly reduced abundance in the mutant compared with parental lines, the most significant in every case were the vicilin proteins which corresponded to the deletions (Table 2, Quad 1; Table 3, Quad 2; Table 4, Quin, TMT; Table 5, Quin, iTRAQ). Notably, the number of proteins in this group was higher than the number of significantly higher abundance proteins in every case (52 versus 18 for Quad 1; 65 versus 17 for Quad 2; 65 versus 29 for Quin TMT; and 54 versus 41 for Quin iTRAQ; Tables 2–5). However, there were other consistent differences apparent among the mutants, in particular a set of ribosomal proteins: 60S ribosomal proteins L7a, L10, L13a-4, and L15 and 30S ribosomal protein S11, which were all present in lower abundance at ~0.3–0.5 of the parental line. Several additional classes of proteins were also consistently identified among the lower abundance proteins in all mutants; these included an array of proteins involved in many aspects of metabolism, mainly primary (adenosylhomocysteinase, calcium-transporting ATPase, mitochondrial phosphate carrier protein 3, monosaccharide-sensing protein 2, oxygen-evolving enhancer protein 3, patatin, peptidyl serine alpha-galactosyltransferase, phospholipase D, polygalacturonase, pyruvate decarboxylase, and pyruvate kinase), with others variously involved in secondary metabolism (cytochrome P450 71A1), structural (chlorophyll A-B binding protein and H/ACA ribonucleoprotein complex subunit), and signalling or defence (ARM repeat superfamily protein, BPI/LBP family protein At1g04970, subtilisin-like protease, and transducin beta-like protein 2) roles. The proteins which were consistently and significantly impacted in two or three of the mutants are listed in Supplementary Table S5 (higher abundance) and Supplementary Table S6 (lower abundance). It is notable that lower abundance proteins common to two of the three mutants were, most often, the same for Quad 2 and Quin mutants which share deletions of all but the *VicD* locus (Supplementary Table S6). Any of these deletion mutants could potentially contain deletions of additional genes, other than vicilins, encoding proteins identified as being of reduced abundance in the mutants (see later).

### Developmental analysis of mutant seeds

**Fig. 5.**
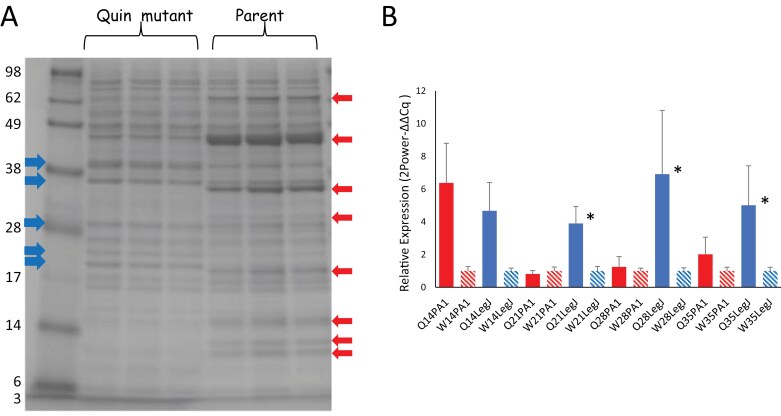
(A) Protein profiles of immature seeds from quintuple mutant and parental lines, at 21 d after flowering. Red arrows highlight the major proteins missing from mutant samples; blue arrows indicate more predominant proteins in the mutant. Protein standards are shown in the left-hand track with sizes indicated (×10^–3^). (B) The expression of *LegJ* (blue) and *PA1* (red) genes in immature seeds from quintuple mutant and parental lines, relative to expression of *EF1α* as the control gene, at four stages of seed development: 14, 21, 28, and 35 d after flowering, as indicated on the *x*-axis; Q and solid colours, Quin mutant; W and hatched colours, control parental line; bars indicate means with SEs (Livak and Schmittgen, 2001). *P=0.000, 0.006, and 0.017 for *LegJ* expression values at 21, 28, and 35 d after flowering, respectively, based on two-sample *t*-tests.

Analysis of protein profiles during seed development emphasized the many differences in seed protein accumulation between the mutant and parental lines, particularly at developmental stages when vicilin synthesis is close to maximal in the control lines. The protein profile of the quintuple and control lines at 21 d after flowering, when additional proteins are evident in the mutant samples, is shown in Fig. 5A. Relative gene expression analysis was carried out for two gene classes: *LegJ* and *PA1 (pea albumin 1)*. Both of these gene classes correspond to proteins which were present at higher abundance in the quintuple mutant (3- to 12-fold change; Tables 4, 5). Analysis at four stages of seed development showed significant differences in relative gene expression for *LegJ* at the later stages analysed (21, 28, and 35 d after flowering), with higher expression in the mutant in every case (Fig. 5B, using *EF1α* as the better matched reference gene); the differences in expression of the target genes were comparable, using either reference gene (not shown).

### A chromosome-scale genome assembly for pea accession JI2822

In order to determine whether some of the proteins (other than vicilins) that were of low abundance in the deletion mutants compared with the wild type (Supplementary Table S6) were encoded by genes within these deletions, we generated a chromosome-scale genome assembly for the parental line, JI2822. We used PacBio HiFi sequencing to establish a megabase-scale draft assembly, and then ordered contigs into chromosome-length scaffolds using chromosome conformation sequencing (Hi-C). In total, we generated 96 Gb of highly accurate long reads with an average read length of 16 kb. This resulted in 2815 contigs with length-weighted median read length (N50) of 4.16 Mb. Hi-C scaffolding resulted in seven chromosomes and 163 unanchored scaffolds, where chromosomes and additional scaffolds comprised a total length of 3.8 Gb (Supplementary Fig. S1; Supplementary Table S3). To assess completeness, we looked for single-copy orthologue genes common in Embryophyta (BUSCO); 99.3% of those genes were found in the JI2822 genome sequence assembly.

We used RNA-seq data from 10 different organs or developmental stages to annotate 33 559 genes. Although the computational gene annotation pipeline detected all vicilin loci in the genome, the precise structure of the *VicA*, *VicB*, and *VicC* genes was manually curated, which mainly involved removal of artificial splice junctions between highly similar adjacent paralogues.

### Analysis of the gene content within the genomic deletions

**Fig. 6.**
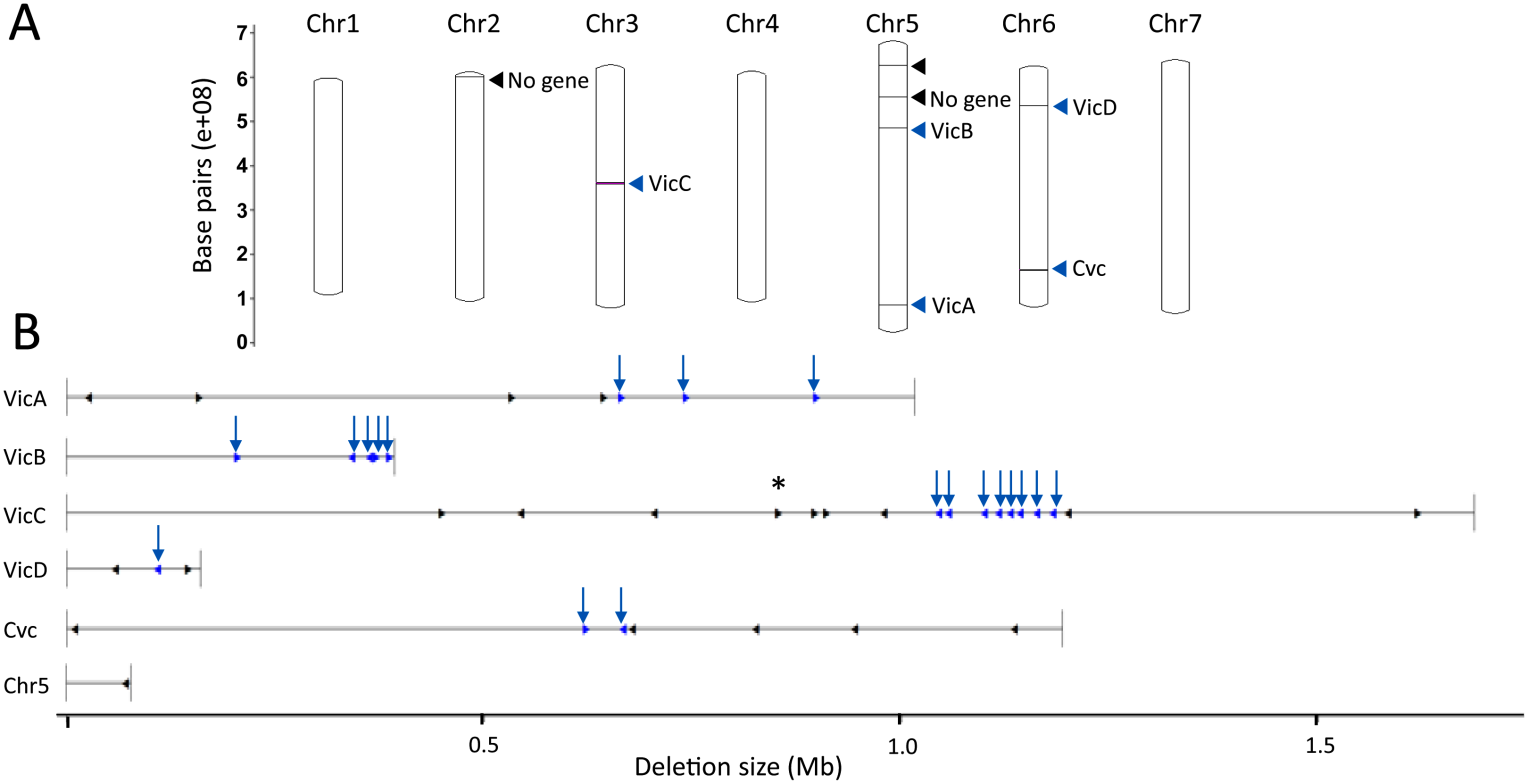
(A) The chromosomal (Chr1-Chr7) positions of the deletions detected in the quintuple mutant determined by sequencing the mutant and parental lines; targeted vicilin and convicilin loci, blue arrows; three additional deletions, black arrows. (B) Scale representation of the deletions in the quintuple mutant encompassing the five vicilin/convicilin loci; genes encoding vicilin or convicilin, blue horizontal arrows, highlighted with blue downward pointing arrows; additional genes, black horizontal arrows. Asterisk indicates two closely positioned subtilase genes (Psat.JI2822.v1.3g034080 and Psat.JI2822.v1.3g034100, Supplementary Table S7) in the mutant lacking *VicC* genes.

The limits of the deletions that include the vicilin and convicilin genes were determined by re-sequencing the genome of the quintuple mutant in order to understand the extent to which the documented protein changes reflected pleiotropic consequences of the loss of certain vicilin proteins from seeds or were a direct consequence of the loss of additional genes within deleted regions. By mapping reads to the genome of the parental line JI2822, we detected eight deletions, the details of which are provided in Supplementary Table S7. The locations of the eight deletions involving chromosomes 2, 3, 5, and 6, and the gene arrangements within each deletion are shown in Fig. 6A and B. The eight deletions include the five involving vicilin and convicilin loci (*ΔVicA–D*, *ΔCvc* in Supplementary Table S7), plus three others (Fig. 6A). The chromosomal positions of the deletions shown in Fig. 6A can be related to those of well-documented genes and traits in pea, both of which have been positioned on the physical map of JI2822, orientating the position of these deletions with respect to genes corresponding to phenotypic markers and chromosomal features on the pseudomolecules (Supplementary Dataset S1). Of the three non-targeted deletions (Fig. 6A), which are unlinked to any of the vicilin or convicilin loci, two impact chr5 (deletions of ~5 kb and 138 kb) and one impacts chr2 (deletion of ~17 kb); only one of these three deletes a predicted gene (encoding a putative disease resistance protein; Supplementary Table S7). In the case of the *ΔVicA*, *ΔVicC*, and *ΔCvc* genomic deletions, the additional genes annotated within each deletion fall outside the vicilin/convicilin genes which are clustered. Genes encoding VicA proteins show the same direction of transcription in every case, as do those for VicC (Fig. 6B). Two genes encoding VicB are in the opposite orientation to the other three, while the two genes encoding Cvc lie in an opposing orientation to each other (Fig. 6B; Supplementary Table S7).

Sequence analysis revealed that the genomic region impacted by the *VicA* gene deletion (*ΔVicA*) included, in addition to three VicA-encoding genes, four different genes, encoding a glycine-rich protein, a putative serine esterase, an epidermal patterning factor-like protein, and one non-annotated gene (Supplementary Table S7). Analysis of the *ΔVicB* deletion revealed that five *VicB*-encoding genes had been deleted but no additional gene was absent. In contrast, the *ΔVicC* deletion impacted eight functional genes encoding VicC (an additional three were predicted to be pseudogenes) plus a further 10 genes, encoding six protein types [Golgin candidate, a RING-H2 finger protein, subtilase-related proteins (three genes), wall-associated receptor kinase-like (two genes), a T cell tolerance induction protein, and pentatricopeptide repeat-containing proteins (two genes)] (Fig. 6B; Supplementary Table S7). For *ΔVicD*, the relatively small deletion has impacted the gene encoding VicD, plus two others, one of which encodes a zinc-finger protein, while the other is not annotated (Supplementary Table S7). Finally, analysis of the *Cvc* deletion revealed that an additional five genes had been deleted; four of these were not annotated, while the fifth encodes the transcription factor implicated in the shape of the keel petals (Wang *et al.*, 2008). All *Cvc* deletion mutants in this study showed the reduced wing petal phenotype of a *k* mutant.

The proteins encoded by these additional (non-vicilin-encoding) genes which had been deleted were not featured among the list of those which were significantly reduced in abundance in the seed proteome of the mutants analysed.

Both the proteomic and genomic sequence data revealed one additional functional vicilin gene (*VicE*), that lies outside the loci previously described. Besides *VicA–E*- and *Cvc*-coding sequences, we checked whether any further genes closely related to vicilins might be expressed in JI2822. One transcript (PsatJI2822.v1.5g39900, gene locus on chromosome 5), which showed 72% DNA sequence identity to *VicA*, *VicB*, and *VicE* and 80% identity with a vicilin-like transcript from *Vicia villosa* (NCBI XM_058926651), was detected. PsatJI2822.v1.5g39900 predicts a truncated protein of 139 amino acids, compared with 459 amino acids for VicB and 461 for the *V. villosa* vicilin-like protein; the protein sequence alignment includes a deletion of 13 amino acids and a loss of the VicB predicted signal cleavage site (data not shown). The gene corresponding to Psat.JI2822.v1.5g39900 (chr5: 188 940 406) lies close to the position of the *ΔVicB* deletion. Analysis of the JI2822 genome sequence revealed all the vicilin genes documented above plus one additional pseudogene, which was not evident in the expressed sequence data. This sequence (start codon at chr3: 196 454 348) lies near the position of the *ΔVicC* deletion and has a single nucleotide polymorphism (T:G) which predicts an N-terminal peptide of 42 amino acids followed by an early stop codon.

### Impact of the combined mutations on protein and amino acid content

**Fig. 7.**
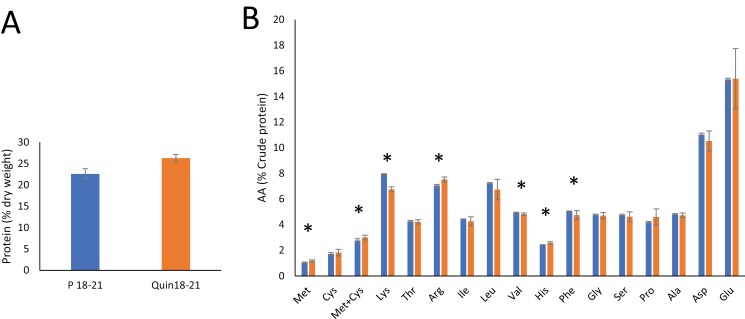
(A) Protein concentration (% dry weight) in mature seeds from quintuple (Quin) mutant and parental (P; JI2822) lines grown under field conditions, determined by the Dumas method. 18–21 refers to combined years of field trials (2018, 2019, and 2021), bars, SEM, *n*=9; n. s. (*P*=0.05, one-way ANOVA). (B) Amino acid concentrations (% protein) in seeds from quintuple mutant and parental lines. Aspartic and glutamic acid data refer to the sum of Asp/Asn and Glu/Gln, respectively; tyrosine and tryptophan were not measured. Seeds were harvested from three microplots grown in each of the three seasons; bars, SEM. Asterisks indicate significant differences (*P*<0.05, one-way ANOVA). Blue, parental line; orange, quintuple mutant.

Amino acid analysis of the mutant lines was carried out using protein hydrolysis and chemical analysis methods developed for use in pea and based on sampling new varieties annually (Evonik Industries AG). Seeds were harvested from field experiments for amino acid analysis. In comparisons of the quintuple mutant and parental lines, there were significant increases in the concentration of 14 individual amino acids in the mutant, when expressed on a dry matter basis; mutant seeds also had a significantly higher dry matter content (*P*<0.01; Supplementary Fig. S6). The increases on a dry matter basis reflected, to a large extent, the higher protein concentration in the mutant seeds (*P*<0.001; Fig. 7A). On a unit protein basis, significant differences in six amino acids were evident; three of these were present at higher concentrations in the parental line (Lys, *P*<0.001; Val, *P*<0.01; Phe, *P*<0.05), whereas three were present at higher concentrations in the mutant seeds (Met, *P*<0.005; His, *P*<0.001; Arg, *P*<0.001) (Fig. 7B). Analysis of Quad 1 and Quad 2 mutant seeds also revealed a higher protein concentration in the mutant lines (*P*<0.001), and significant differences in amino acid concentrations that were similar to those observed for the quintuple mutant. Consistently higher concentrations of methionine, histidine, and arginine were evident in both mutants, while lysine and phenylalanine were consistently higher in the parental line (Supplementary Fig. S7). However, some differences were apparent between the two quadruple mutants including, for example, valine, glycine, and proline which were differentially affected in the two quadruple mutants when compared with the parental line.

### Impact of the combined mutations on starch-related metabolites and seed yield

**Fig. 8.**
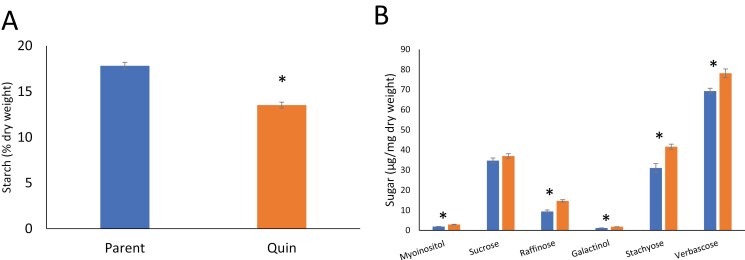
(A) Starch concentration (% dry matter) in mature seeds from the quintuple mutant (Quin) compared with the parental line, based on replicate plots from one season (*n*=3; *P*<0.001, one-way ANOVA). (B) Concentration of six metabolites in seeds from quintuple mutant and parental lines. Asterisks indicate significant differences (*P*<0.005); *n*≥3, bars, SEM, one-way ANOVA. Blue, parental line; orange, quintuple mutant.

Comparisons of the quintuple mutant and parental lines, when total seed starch and a series of metabolites related to the raffinose oligosaccharide pathway were quantified, are shown in Fig. 8. Starch concentrations per unit dry weight in mature seeds were decreased significantly in the mutant (*P*<0.001), while all other metabolites measured, except for sucrose, were significantly increased in the mutant (*P*<0.005; Fig. 8A, B). The same changes in these metabolites were apparent in comparisons of the Quad 1 and Quad 2 mutants with the parental line; in the case of Quad 1, however, sucrose levels were also elevated from ~35 µg mg^–1^ seed meal to 45 µg mg^–1^ (*P*<0.005; data not shown).

**Fig. 9.**
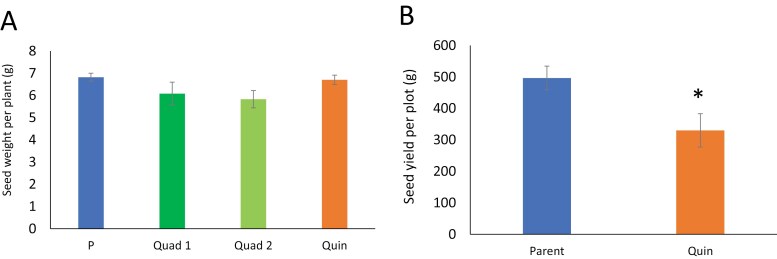
(A) Seed yield (per plant, g) from quadruple (Quad) and quintuple (Quin) mutant and parental (P) lines under greenhouse conditions; bars, SEM, *n*=10, n. s., one-way ANOVA. (B) Seed yield (per plot, g) from Quin and parental lines under field conditions, based on nine plots of each grown over three seasons; an asterisk indicates a significant difference (*P*<0.05, one-way ANOVA). Blue, parental line; orange, quintuple mutant; dark green, Quad 1; light green, Quad 2.

Despite the reduced concentration of starch in their mature seeds (% dry weight, Fig. 8A), mutant seeds were overall slightly larger than those of the parental line, JI2822 [0.176, 0.181, 0.178, and 0.158 g mean seed weights for Quad 1, Quad 2, and Quin mutants, and parental line, respectively (SEM=0.004–0.005, *n*=8–10; *P*<0.01)]. This difference was compensated for by significantly fewer numbers of seeds per plant in the mutants, such that there was no significant difference in the yield of seeds from the quadruple and quintuple mutants when assessed under greenhouse conditions (Fig. 9A). There was, however, a reduced yield in the quintuple mutant line overall, when grown under field conditions over multiple plots and seasons (three plots in each of three seasons; Fig. 9B).

### Impact of the mutations on functional properties of the seed

**Fig. 10.**
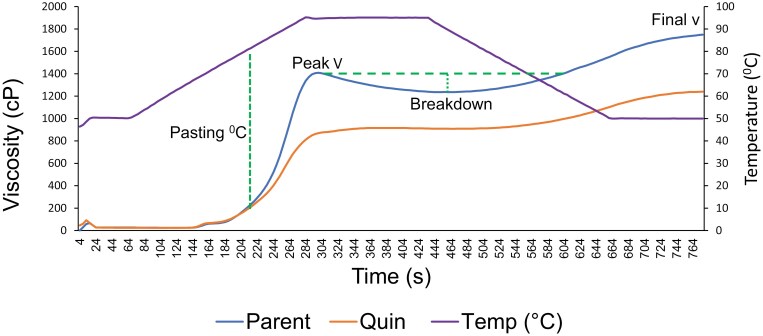
Relative viscosity analysis of seed meals derived from quintuple mutant (red) and parental (blue) lines. Temperature changes are indicated (purple line) in relation to changes in viscosity (v) of the pea samples. The green dotted lines indicate pasting temperature and breakdown parameters. Breakdown is defined as the difference between the peak viscosity and the lowest viscosity during the holding stage, as determined by the shear force and composition of the mixture. Time, seconds; cP, centipoise. Several RVA-associated parameters (peak 1, trough, breakdown, final viscosity, and setback), but not pasting temperature, differed significantly (one-way ANOVA).

Seed meal samples from the quintuple mutant and parental lines were assessed using a relative viscosity analyser (RVA). RVA reflects complex interactions among variable components, chiefly starch, protein, and water, as affected by temperature and time. The profiles obtained for the two lines were significantly different in terms of viscosity and profile shape (Fig. 10). The lower viscosity observed for the quintuple mutant line is likely to be partly a consequence of its lower starch concentration compared with the parental line. A lower breakdown suggests differences in heat stabilities which could be due to changes in protein–starch interactions, reflecting the different protein composition of the lines tested (Fig. 10).

## Discussion

In this paper, we describe the generation and analysis of a set of pea lines, where seeds lack some, or the majority of, the vicilin family of seed proteins, as a consequence of combining up to five FN-derived deletion mutations. This set of lines provides a unique resource, in a genetic background isogenic to the wild type, where the mutations have been described in terms of their impact on the proteome and the corresponding genomic deletions defined precisely, with reference to the high-quality reference genome assembly which has been generated for the parental line. This provides a fourth publicly available chromosome-scale genome assembly for pea, alongside that of the cv. Caméor (Kreplak *et al.*, 2019), ZW6 (Yang *et al.*, 2022), and Zhewan No. 1 (Liu *et al.*, 2024). The present study demonstrates the effectiveness of mutant populations, especially in combination with an available genome sequence. JI2822-derived mutants have been used extensively in previous genetic studies and candidate gene identification (for examples, see Domoney *et al.*, 2013; Tayeh *et al.*, 2024, and references therein). The availability of the parental genome sequence assembly will support and accelerate related research and open up further avenues of research, especially for the analysis and further utilization of the FN mutant population, as described here.

Vicilin mutants are of interest both fundamentally, in terms of questions on dispensability or otherwise of the related protein classes, and for application of pea products in industrial processes. While the present work shows that vicilins are dispensable for pea seed viability, many questions regarding functions *in planta* remain to be addressed. Such studies will be facilitated by the suite of related mutants developed within a common genetic background. The ratio of vicilins to legumins has been shown to influence a wide range of food-related traits, including solubility, emulsification, and foaming in pea (Barac *et al.*, 2010) and in soybean (Pavlicevic *et al.*, 2018). Here we have shown through preliminary analysis of the functional properties of seed isolates from the quintuple mutant that parameters revealed though RVA were impacted (Fig. 10); such variation is sought widely for the development of plant-based foods as substitutes for animal-derived products (Troya *et al.*, 2021; Rogers *et al.*, 2024). Once mutants have been bulked for industrial-scale tests, they can be tested for their impact on traits such as water binding, gelling, foaming, and emulsification. Others have tested the effects of altered protein composition, including altered vicilin:legumin ratios, on a number of industrially relevant processes, with conflicting results (Gravel *et al.*, 2024; Husband *et al.*, 2024). However, none has tested near-isogenic plant materials, or extreme variants such as those described here, where background genetic effects have been minimized. The introduction of the suite of vicilin-related deletion mutations into commercial cultivars offers such an opportunity, assisted by the tools and resources reported here.

The mutant lines identified here carry deletions which include all five genetic loci previously described as encoding vicilins or convicilins, where their discrete sequences and unlinked genetic locations facilitated their genetic combination in this work (Fig. 6; Supplementary Dataset S1). Genomic sequence analysis of the derived quintuple mutant confirmed that, in every case, the deletions included the cluster of vicilin or convicilin genes at each locus, validating the primer-based mutant population screens (Table 1). With the exception of VicD, none of the proteins encoded by the targeted genes has been implicated directly in a biological role beyond that of a seed storage protein function. VicD was previously described as a vicilin-related (Domoney and Casey, 1990), putative sucrose-binding, protein (Domoney *et al.*, 2013), based on the localization of transcripts from the orthologous gene in *Vicia faba* over seed coat vascular strands and the embryonic epidermal transfer cell layer (Heim *et al.*, 2001). It has furthermore been shown, in pea, that the VicD protein (otherwise known as p54) is at least partially processed to yield two proteins of 16 000 Da (from the N-terminus) and 38 000 Da, both of which co-localized at a subcellular level as chromosomal proteins (Castillo *et al.*, 2005); aspects of the regulation of p54 during seed maturation have been reported (Gagete *et al.*, 2011). Such a location is suggestive of a possible role for these proteins in genome stability and chromosome compaction during desiccation (van Zanten *et al.*, 2012).

Furthermore, the *VicD* gene (*pps54*) has been reported to be differentially induced by ABA and other stress situations, suggesting that this gene represents a distinct class of vicilin (Castillo *et al.*, 2000, 2005). However, this latter property may be shared more widely among vicilins and other seed protein genes, given their structural relatedness within the super-family of cupin-related proteins, which includes proteins such as spherulin, a stress-induced protein of the slime mould *Physarum polycephalum*, and others implicated in desiccation tolerance (Dunwell *et al.*, 2000, 2004).

Further roles of vicilins as antimicrobial or defence peptides might be postulated, based on additional studies of related proteins (including those classified as 7S proteins) (Sales *et al.*, 2000; Gomes *et al.*, 2002; Rose *et al.*, 2003). Additionally, vicilin proteins from the closely related legumes (*Lupinus albus*, *Vicia faba*, and *Lathyrus sativus*) have been proposed to behave as lectins, based on their binding affinity for mammalian membranes and elution with appropriate sugars (Ribeiro *et al.*, 2014), a property shared by lectins shown to have defence roles.

The consequences of near abolition of a major class of seed storage protein in pea have been shown here to lead to major changes in the balance of seed protein composition (Figs 2B, 3, 4; Tables 2–5), such that the concentration of total seed protein per unit dry weight in mature seeds is not diminished (Figs 2A, 7A; Supplementary Figs S2A, S5A, S7A). Deployment of an RNAi strategy to suppress the synthesis of soybean (*Glycine max*) major storage proteins has been shown to lead to a rebalancing of protein content with a selective increase in the accumulation of just a few proteins (Schmidt *et al.*, 2011). In *Phaseolus*, lines lacking certain seed proteins have also been generated (Taylor *et al.*, 2008; Campion *et al.*, 2009). Total seed protein content was not impacted by the loss of phaseolin, phytohaemagglutinin, and arcelin in *Phaseolus vulgaris*. Reductions in the amount of a glutamyl dipeptide of *S*-methyl-cysteine, accumulated in high concentrations in this species, were noted in the mutant lines, but it should be noted that the lines being compared were not near-isogenic (Taylor *et al.*, 2008). Our analysis of the pea deletion mutants (quadruple and quintuple vicilin/convicilin null combinations) has shown that a wide set of changes occurs in the mutants, affecting many proteins which are increased or reduced in abundance. It is particularly noteworthy through both qualitative gel and quantitative proteomic analyses that the legumin protein class has been significantly impacted, with the LegJ class shown to be elevated in the mutants analysed. Although this might be expected to result in a notable shift in the amino acid profile of the corresponding seeds, in particular in the sulfur-containing amino acids which are more prevalent in legumins than vicilins, in fact only small differences in methionine content are observed (Fig. 7B; Supplementary Figs S6B, S7B). Presumably this is a consequence of the reduced accumulation of additional proteins beyond vicilins, which are synthesized from a limited pool of amino acids. Besides legumins and an uncharacterized protein, four further proteins were present in higher abundance in the three mutant combinations: a BURP domain-containing protein, two enoyl reductase (ER) domain-containing proteins and a PXR1-like protein, with others common to two of the mutant combinations. Albumin 1, a protein with a high concentration of sulfur-containing amino acids (Eyraud *et al.*, 2013), was present in higher abundance in the quintuple mutant only. It should be noted also that the proteomic and genomic data revealed an additional hitherto unidentified vicilin protein (named VicE) and corresponding gene locus with, unusually, a single gene copy; the corresponding protein was increased in abundance in two of our mutant combinations (Quin and Quad 2, the latter of which carries deletions of all vicilin/convicilin targets except for *VicD*) (Tables 1, 3–5; Supplementary Table S5).

Of the proteins which were consistently of lower abundance in mutants, none corresponded to those few genes which were identified amongst the annotated genes within the deletion intervals; we conclude that these additional genes do not make a significant contribution to seed proteins (Tables 2–5; Supplementary Tables S6, S7). A set of ribosomal proteins is of particular note within the group of lower abundance proteins in mutants: 60S proteins L7a, L10, L13a-4, and L15, with additional ribosomal proteins impacted in the quadruple mutants (Supplementary Table S6). The impact of this effect is unlikely to be reflected in changes in translational activity within the seed *per se*, since the overall accumulation of seed proteins is unimpaired. However, as the seed proteome reflects the totality of proteins accumulated during seed development, it may be that, during the period of vicilin accumulation in seeds (early-mid stages), there is reduced demand for ribosomal proteins during that time, and the later period of protein synthesis does not compensate entirely for this. However, our data suggest large differences in the expression of legumin genes at mid to late developmental stages, as measured by qPCR (Fig. 5B) and suggested by the proteomic analyses (Tables 2–5). An overall effect on ribosomal activity would be expected to impact thirty-three 40S and forty-seven 60S ribosomal proteins, rather than the subset of 12 we observed. Alternatively, and more plausibly perhaps, some ribosomal proteins have been implicated in extra-ribosomal functions and developmental processes, including miRNA biogenesis, anti-virus defence, and plant immunity (Yan *et al.*, 2016; Martinez-Seidel *et al.*, 2020; Xiong *et al.*, 2021). Among the ribosomal proteins impacted in all three vicilin mutant combinations, L10 may be of particular interest, with its reported involvement in the transcriptional regulation of certain genes (Xiong *et al.*, 2021). However, it should be noted that the annotation and naming of such proteins is generally problematical (Lan *et al.*, 2022) and, as such, apparent identities need to be treated with caution.

Finally, the allergenic potential of vicilin proteins is related to their structures as bicupins (Dunwell *et al.*, 2004; Robinson *et al.*, 2022). The mutant lines described here offer the potential to develop and test food products with a greatly diminished allergenic potential, based on reported dose responses (Taylor *et al.*, 2021). The possible plant defence roles of vicilin protein may be met in such materials by the retention of the minor single gene-encoded variant vicilin, VicE, identified through this work, as well as through retention of the stress-responsive VicD (see above). In such mutants, two of the 20 functional vicilin-related genes (Table 1; Fig. 6B) would be retained, and hypothesized to resolve conflicting plant and nutritional roles. Of these two, the mature VicD protein is notably distinct from the other vicilin proteins, showing, for example, 27% and 26% identity (46% similarity) to VicA and VicB, respectively, compared with values of 81% (91%) for a comparison of VicA with VicB.

Overall, the genetic materials provide much opportunity for further research into the functional properties of vicilins. The data presented suggest that there may be a yield penalty associated with quintuple vicilin mutants under field conditions, but introgression into a more agronomically adapted genetic background would be needed to substantiate this conclusion. JI2822 is a dwarf pea accession, developed for laboratory studies and poorly adapted to field studies; its wrinkled seeds, due to the *rb* mutation impacting starch biosynthesis (Rayner *et al.*, 2017), lead to sensitivities to water uptake by germinating seeds, which is more apparent in cold field conditions. Introgression of the vicilin mutations into a commercial background would be expected to compensate for the yield penalty caused by the *rb* mutation, the impact of which might be exacerbated by the lack of vicilin proteins during development. Back- and inter-crossing single vicilin locus mutants would eliminate any possible impact from the additional deletions (Fig. 6; Supplementary Table S7) and could reveal the genetic determinants of the responses to different growth environments.

The impact of the environment on seed protein composition is clear from research in many species (Tabe *et al.*, 2002; Bourgeois *et al.*, 2009; Cartelier *et al.*, 2021), most notably where the synthesis of sulfur amino acid-containing proteins is dramatically reduced under sulfur-limiting conditions (Casey and Domoney, 1999; Shewry and Casey, 1999). In pea, such a deficiency leads to the maintenance of vicilin synthesis over a longer developmental period, reflecting transcriptional regulation (Beach *et al.*, 1985). Together with the genetic effects described in the present work, we may conclude that seed protein synthesis demonstrates a high degree of plasticity in adapting to available pools of amino acids; quantitative differences in expression of *LegJ* shown here in the quintuple mutant are consistent with transcriptional changes.

It is possible that the quadruple mutant, Quad 2 (having *VicD* present, encoding a putative sucrose-binding/stress-responsive protein), may offer the best prospects for a breeding programme. *VicD* is genetically very close to the gene controlling anthocyanin pigmentation in pea (*A*; Hellens *et al.*, 2010, where *VicD* is referred to as CD72); where desired, recombination between *VicD* and *A* (2 Mb apart in the JI2822 genome) could be achieved, predicted at a rate of 1–2% based on the JI0281×Caméor population genetic map (Ellis *et al.*, 2023). The genetic background of the mutant line is purple-flowered, with coloured testa (*AA*), whereas most commercial breeding programmes require white flowers and a transparent seed testa (*aa*).

## Supplementary data

The following supplementary data are available at *JXB* online.

Fig. S1. A Hi-C contact heatmap, showing interaction frequency of Hi-C data on JI2822 chromosomes 1–7.

Fig. S2. (A) Protein concentration in mature seeds of mutants identified as lacking individual vicilin loci compared with the parental line, JI2822. (B) Samespots alignment of a 2D analysis of seed proteins from a mutant identified as lacking *VicB* genes and JI2822.

Fig. S3. Samespots alignment of a 2D analysis of seed proteins from a mutant identified as lacking *VicC* genes and JI2822.

Fig. S4. Samespots alignment of a 2D analysis of seed proteins from a mutant identified as lacking *VicD* genes and JI2822.

Fig. S5. (A) Protein concentration in mature seeds of a mutant identified as lacking convicilin (*Cvc*) genes compared with the parental line, JI2822. (B) Samespots alignment of a 2D analysis of seed proteins from a mutant identified as lacking *Cvc* genes and JI2822.

Fig. S6. (A) Dry matter determinations for mature seeds from quintuple mutant and parental lines, prior to amino acid analyses. (B) Amino acid concentrations in seeds from quintuple mutant and parental lines.

Fig. S7. (A) Protein concentration in mature seeds from quadruple mutant and parental lines, determined by the Dumas method. (B) Amino acid concentrations in seeds from quadruple mutant and parental lines.

Table S1. List of primers used for the detection and characterization of vicilin deletion mutations.

Table S2. Identities of the samples labelled with iTRAQ and TMT for proteomic analysis.

Table S3. Summary statistics for the genome assembly of JI2822, generated from PacBio Hi-Fi and Hi-C sequencing.

Table S4. Summary data for the 2D quantitative gel analyses (Spotpix data) of the single deletion mutants lacking genes encoding VicA, VicB, VicC, VicD, and Cvc.

Table S5. Summary of proteins which are present in higher abundance in the quadruple and quintuple null mutants compared with the parental control.

Table S6. Summary of proteins which are present in lower abundance in the quadruple and quintuple null mutants compared with the parental control.

Table S7. Summary of the functional genes which have been identified as deleted in the quintuple mutant, as determined by genome sequencing of the mutant and parental line.

Protocol S1. Repeat-masking methodology.

Dataset S1. Physical map of JI2822, showing precise location of deletions present in the quintuple vicilin null mutant with respect to genes corresponding to phenotypic markers, well-documented genes, and traits in pea.

erae518_suppl_Supplementary_Figures_S1-S7_Protocol_S1_Dataset_S1

erae518_suppl_Supplementary_Tables_S1-S7

## Data Availability

The mass spectrometry proteomics data have been deposited to the ProteomeXchange Consortium via the PRIDE partner repository (Perez-Riverol *et al.*, 2016, 2022; Deutsch *et al.*, 2023). The dataset identifiers are PXD048561 for the TMT labelling and PXD048566 for the iTRAQ labelling. All the pea genome sequence and associated data are available *via* the Zenodo repository (doi: 10.5281/zenodo.12755082). The raw data and final genome assembly for JI2822 are available through EMBL-EBI (Study ID PRJEB75659 and GCA_964186695, respectively).
