# Supplementary material for: Rebalancing the seed proteome following deletion of vicilin-related genes in pea (Pisum sativum L.)
Source: J Exp Bot. 2024 Dec 21;76(20):5830–60. doi: 10.1093/jxb/erae518 (PMC12621101; doi:10.1093/jxb/erae518)
Supplement: erae518_suppl_Supplementary_Figures_S1-S7_Protocol_S1_Dataset_S1 [file erae518_suppl_supplementary_figures_s1-s7_protocol_s1_dataset_s1.pdf]

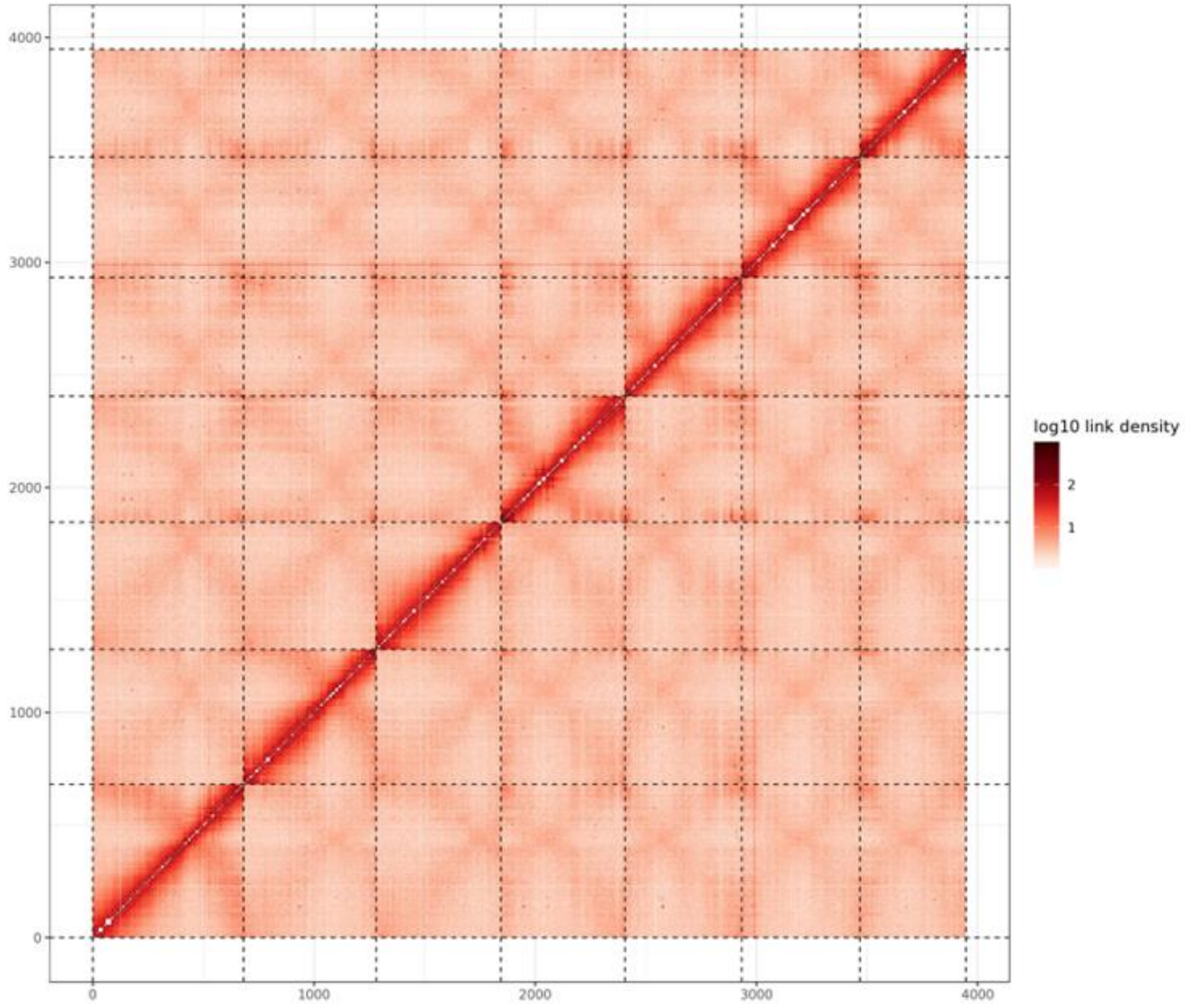

Supplementary Figure S1. A Hi-C contact heatmap, showing interaction frequency of Hi-C data on JI2822 chromosomes 1 to 7 (left to right and bottom to top squares, respectively). Numbers, Mb pairs.

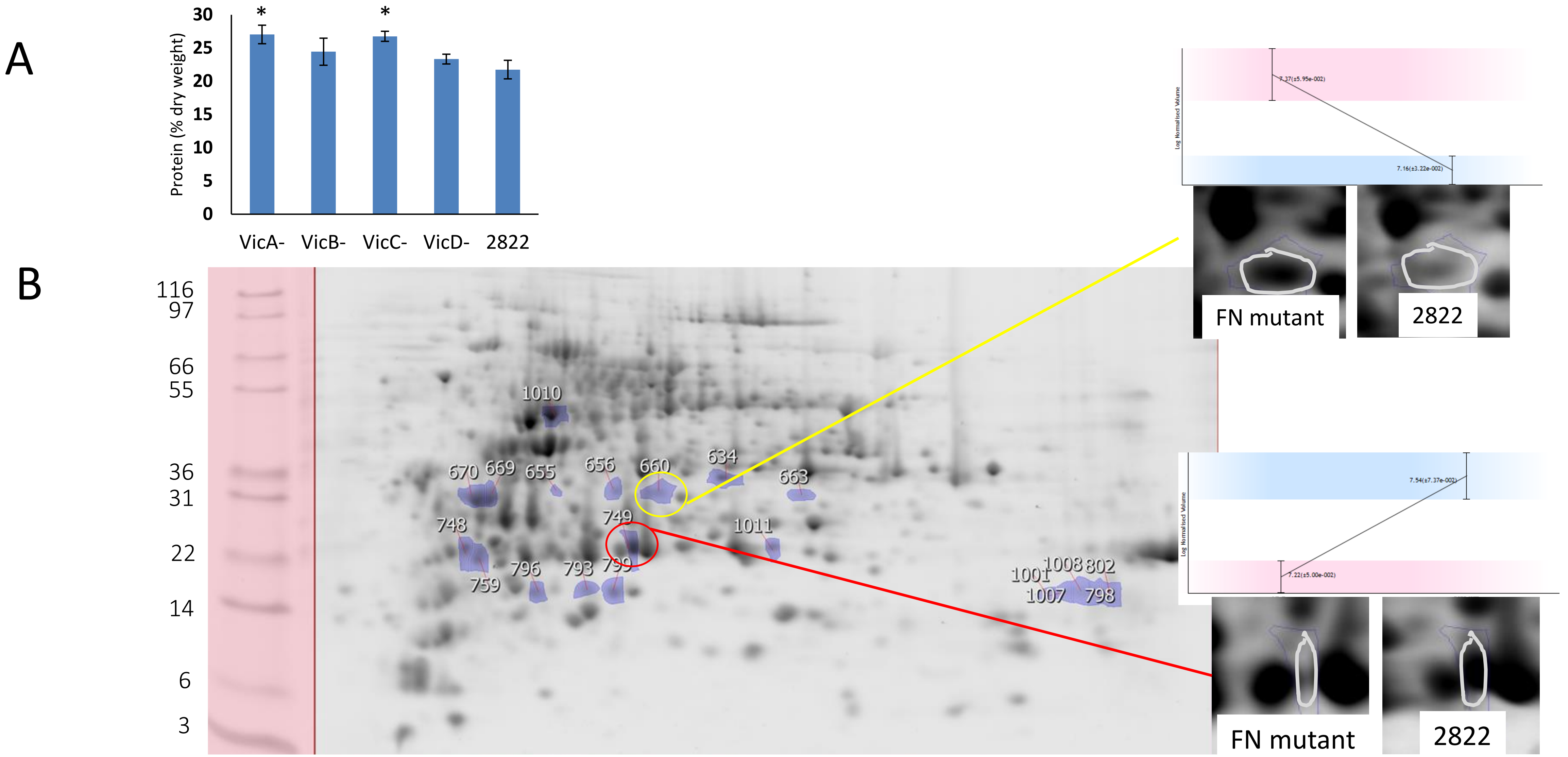

Supplementary Figure S2. A: Protein concentration (% dry weight) in mature seeds of mutants identified as lacking individual vicilin loci (*VicA*, *VicB*, *VicC*, *VicD*) compared with the parental line, JI2822; bars, standard error of the mean, n=6, **P*<0.01 (t-test). **B:** Samespots alignment of a two-dimensional analysis of seed proteins from a mutant identified as lacking *VicB* genes (FN3272/1675_1) and JI2822. All significant differences are coloured (purple). Examples of proteins showing significant differences in abundance are highlighted by circles (VicB, red; VicA, yellow). Details of the two differential regions highlighted are shown, along with their quantification data (log normalised spot volume, FN mutant FN3272/1675_1, left; JI2822, right). Molecular weight markers are indicated on the left (x10^{-3}).

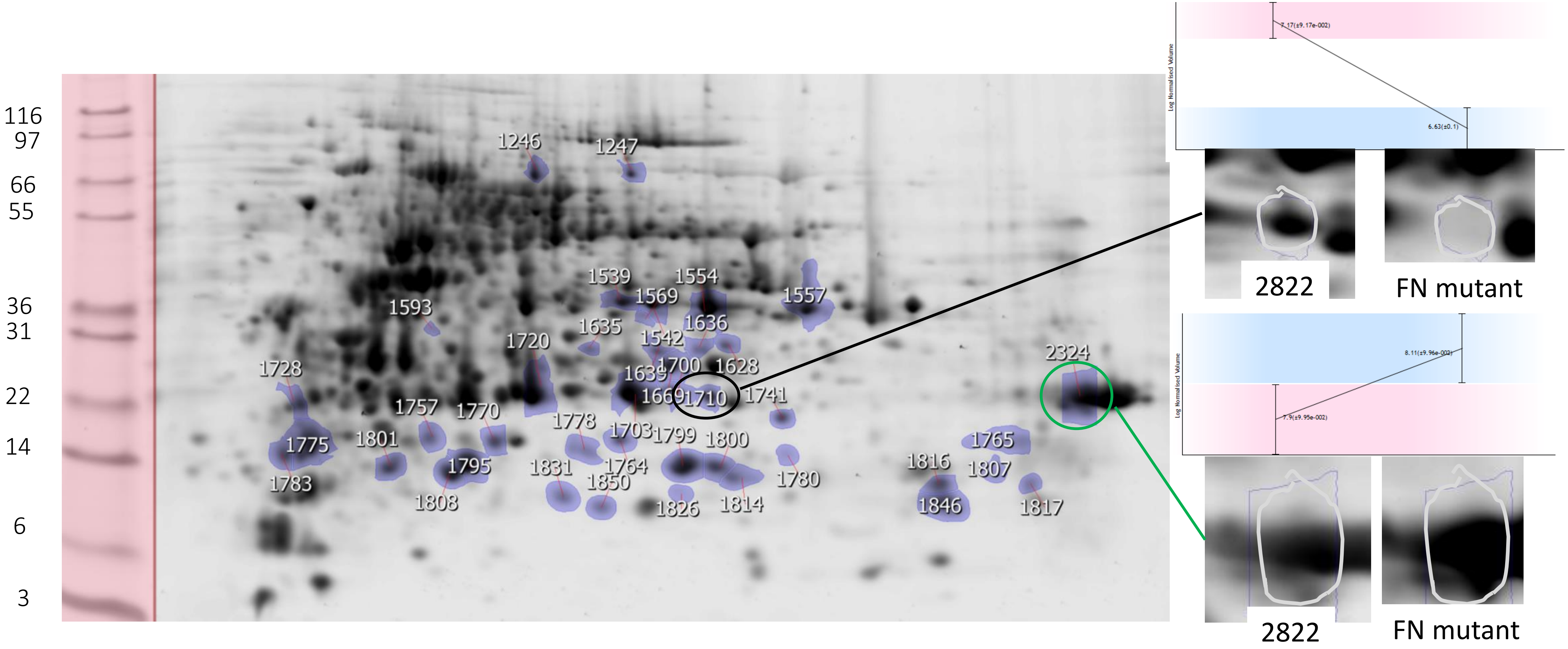

Supplementary Figure S3. Samespots alignment of a two-dimensional analysis of seed proteins from a mutant identified as lacking *VicC* genes (FN1207/2_1) and JI2822. All significant differences are coloured (purple). Examples of proteins showing significant differences in abundance are highlighted by circles (VicC, black; Legumin, green*)*. Details of the two differential regions highlighted are shown, along with their quantification data (log normalised spot volume, JI2822, left; FN mutant FN1207/2_1, right). Molecular weight markers are indicated on the left (x10^{-3}).

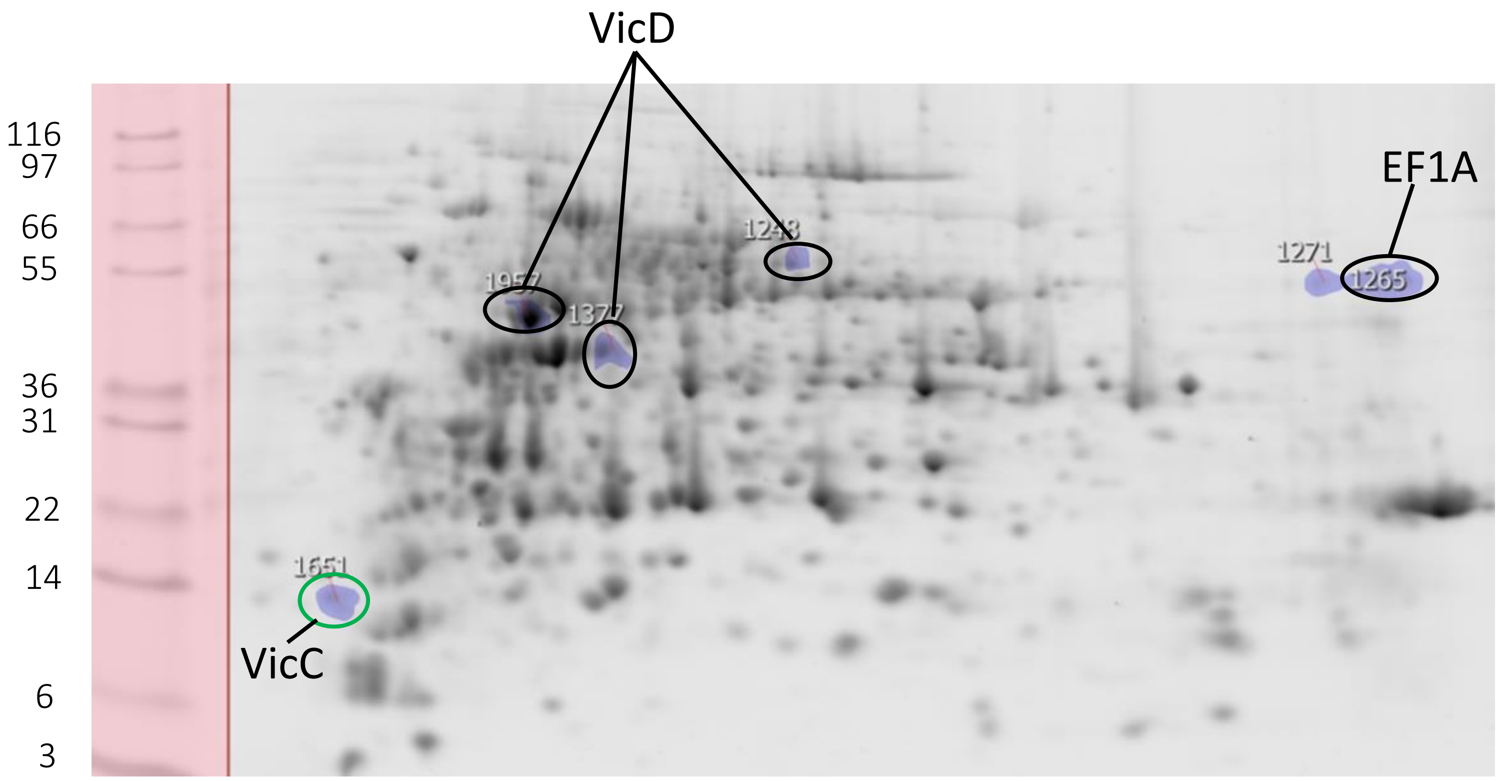

Supplementary Figure S4. Samespots alignment of a two-dimensional analysis of seed proteins from a mutant identified as lacking *VicD* genes (FN3534/2653_1) and JI2822. Six significant differences are coloured (purple), of which five were identified, as shown. Four showed a lower abundance in the mutant and one a higher abundance (green circle). Molecular weight markers are indicated on the left ($x10^{-3}$).

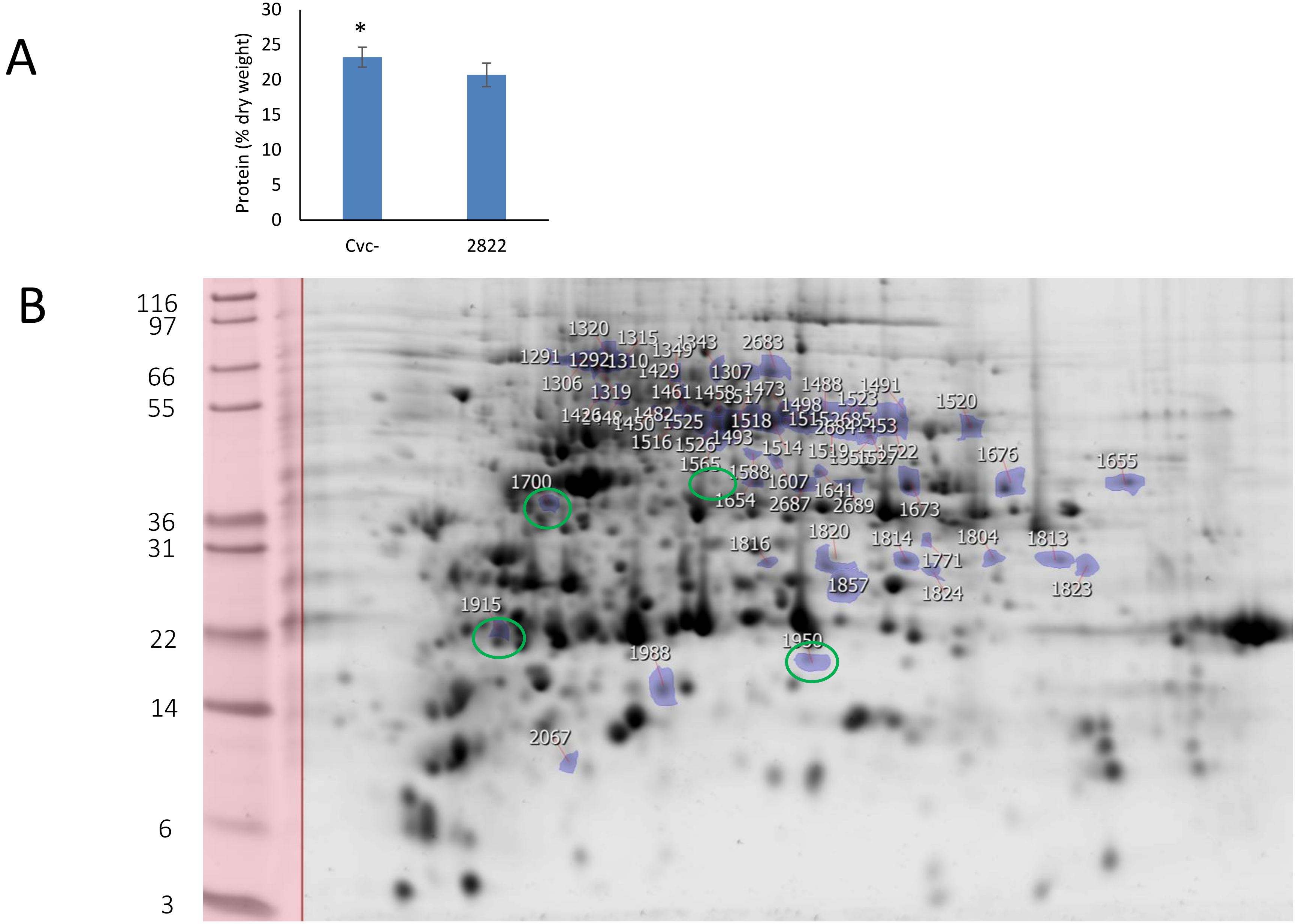

Supplementary Figure S5. A: Protein concentration (% dry weight) in mature seeds of a mutant (FN3082/905_1) identified as lacking convicilin (*Cvc*) genes (left) compared with the parental line, JI2822 (right); bars, standard error of the mean, n=4, **P*<0.01 (t-test). **B:** Samespots alignment of a two-dimensional analysis of seed proteins from FN3082/905_1 and JI2822. All highly significant differences are coloured (purple). Four proteins which showed higher abundance in the mutant line are indicated (green circles), of which three corresponded to VicA.

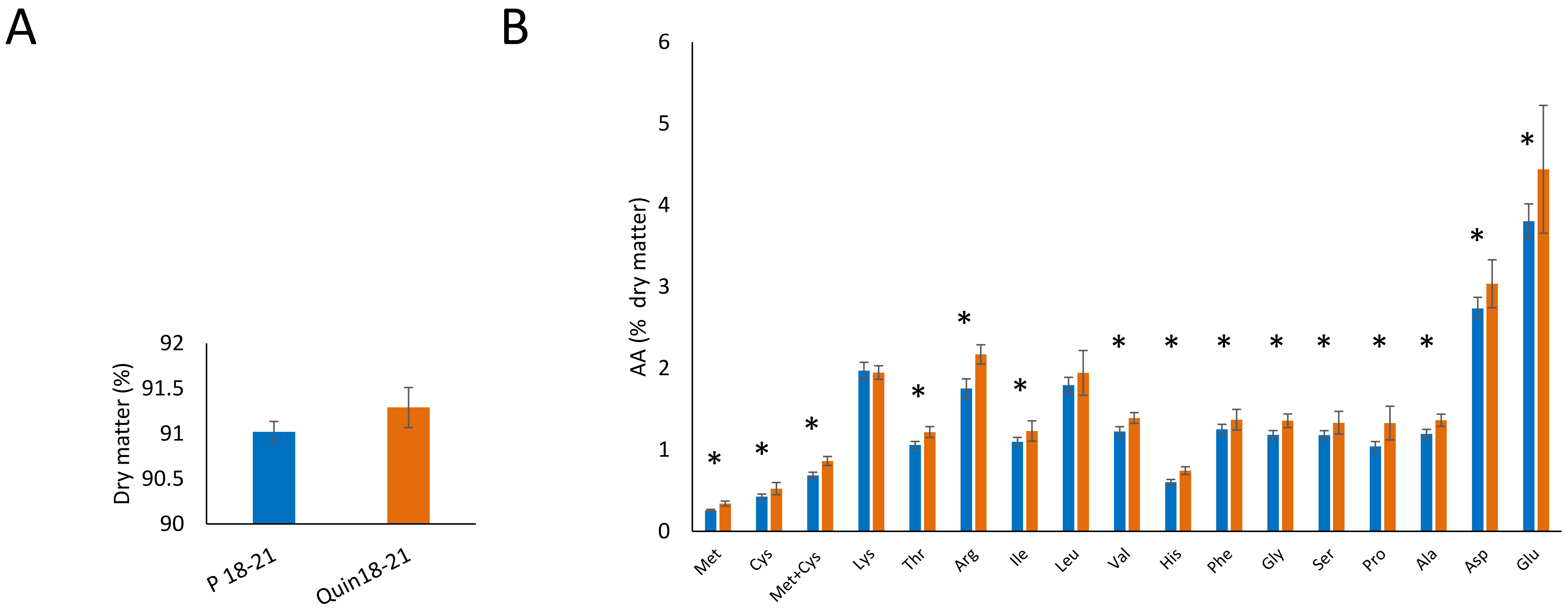

	Met	Cys	Met+Cys	Lys	Thr	Arg	Ile	Leu	Val	His	Phe	Gly	Ser	Pro	Ala	Asp	Glu
ANOVA P	0.000 *	0.002 *	0.000 *	0.595	0.000 *	0.000 *	0.010 *	0.144	0.000 *	0.000 *	0.022 *	0.000 *	0.008 *	0.001 *	0.000 *	0.013 *	0.032 *

Supplementary Figure S6. A: Dry matter determinations (% dry weight, before drying at 103ºC) for mature seeds from quintuple mutant (Quin) and parental (P) lines, prior to amino acid analyses, 18-21 refers to combined years of field trials (2018, 2019, 2021); bars, standard error of the mean, n=9, n. s. (*P*=0.05, one-way ANOVA). **B:** Amino acid (AA) concentrations (% dry matter) in seeds from quintuple mutant and parental lines. Aspartic and Glutamic acid data refer to the sum of Asp/Asn and Glu/Gln, respectively; tyrosine and tryptophan, not measured. Seeds were harvested from three microplots grown in each of three seasons; bars, standard error of the mean. Asterisks indicate significant differences, shown in the table beneath (*P*, one-way ANOVA). Blue, parental line; orange, quintuple mutant

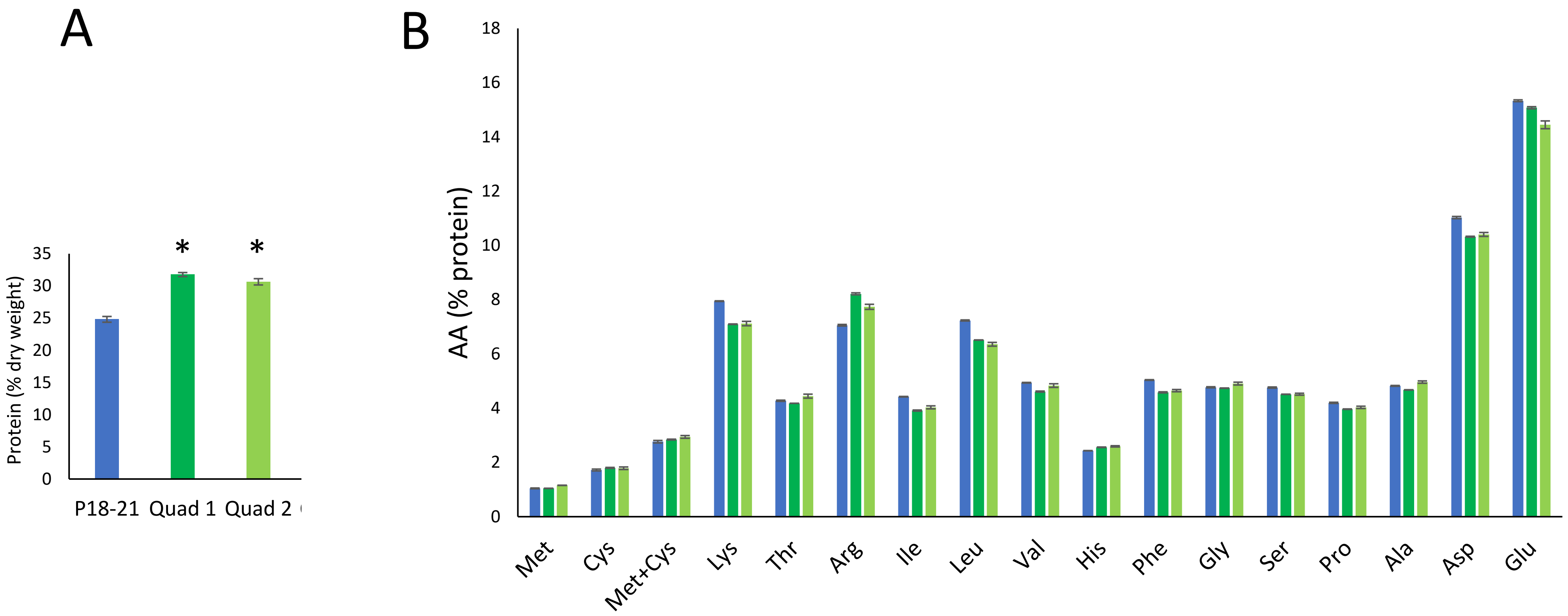

All data	dry matter	crude P	Met	Cys	Met+Cys	Lys	Thr	Arg	Ile	Leu	Val	His	Phe	Gly	Ser	Pro	Ala	Asp	Glu
Quad 1	0.000	0.000	0.012	0.001	0.000	0.000	0.001	0.000	0.002	0.000	0.001	0.006	0.000	0.929	0.000	0.000	0.000	0.000	0.012
Quad 2	0.009	0.000	0.000	0.037	0.003	0.001	0.065	0.002	0.002	0.000	0.227	0.002	0.001	0.035	0.002	0.043	0.029	0.002	0.005

Supplementary Figure S7. A: Protein concentration (% dry weight) in mature seeds from quadruple mutant (Quad 1 and Quad 2) and parental (P) lines, determined by the Dumas method. 18-21 refers to combined years of field trials (2018, 2019, 2021), bars, standard error of the mean, n=9, 3 and 6 for parental, quadruple 1 and quadruple 2 samples, respectively; P<0.001, one-way ANOVA. **B:** Amino acid concentrations (% protein) in seeds from quadruple mutant and parental lines. Aspartic and Glutamic acid data refer to the sum of Asp/Asn and Glu/Gln, respectively; tyrosine and tryptophan, not measured. Seeds were harvested from three microplots grown in each of one (Quad 1), two (Quad 2) or three (parental line) seasons; bars = standard error of the mean, P values are given in the table beneath (two-sample t-tests). Blue, parental line; dark green, Quad 1; light green, Quad 2

Supplementary Protocol S1. Repeat masking methodology

Tandem repeats were detected with TideHunter (Gao et al. 2019) and annotated with TideCluster. TideCluster is a wrapper that runs TideHunter and processes its output to provide comprehensive information about the most abundant families of tandem repeats in the analyzed genome (https://zenodo.org/doi/10.5281/zenodo.7885625). Tandem repeats with a monomer size of 40 bp to 3 kb and a minimum array length of 5 kb were annotated using the default settings of TideCluster. Repeats with a monomer size between 10 and 39 bp and a minimum array length of 5 kb were identified using the TideCluster parameters -T"-p 10 -P 39 -c 5 -e 0.25" -m 5000.
Sequences encoding conserved protein domains of transposable elements were annotated using the DANTE pipeline. DANTE results were then analyzed with DANTE_LTR to annotate full-length copies of LTR- retrotransposons (Novak et al., 2024; Neumann et al., 2019). DANTE and DANTE_LTR were run on the RepeatExplorer Galaxy server (https://repeatexplorer-elixir.cerit-sc.cz/).
A reference library of repetitive elements was constructed from consensus sequences of identified tandem repeat families and representative sequences of full-length LTR-retrotransposons selected from the output of DANTE_LTR using the script "dante_ltr_to_library" (available at https://github.com/kavonrtep/dante_ltr). The library was supplemented with sequences of LINE elements, class II transposons and rRNA gene arrays from our proprietary pea repeat database. The library was used as a reference for the RepeatMasker annotation (Smit et al., 2013-2015) of the JI2822 assembly. The RepeatMasker search was performed on the RepeatExplorer Galaxy server with the options "-xsmall -no_is -e ncbi". Assembly regions with detected similarity to mobile elements that overlapped with tandem repeats annotated by TideCluster were removed from the annotation using bedtools (Quinlan and Hall, 2010) with the command “bedtools subtract”. The resulting GFF3 file was then merged with the DANTE annotation using a custom R script (https://github.com/kavonrtep/granges_tools). The classification of the mobile elements in the annotation files corresponds to the classification system used in the REXdb database (Neumann et al., 2019). For the final repeat-masking, all the above GFF3 files were consolidated by merging the annotated regions into a single BED file using the bedtools merge tool (Quinlan and Hall, 2010). All associated files are available in Zenodo repository 10.5281/zenodo.12755082.

Gao, Y., Liu, B., Wang, Y., Xing, Y. (2019) TideHunter: efficient and sensitive tandem repeat detection from noisy long-reads using seed-and-chain. Bioinformatics 35, i200–i207, https://doi.org/10.1093/bioinformatics/btz376

Novák, P., Hoštáková, N., Neumann, P. and Macas, J. (2024) DANTE and DANTE_LTR: lineage-centric annotation pipelines for long terminal repeat retrotransposons in plant genomes. NAR Genomics and Bioinformatics 6:113. https://doi.org/10.1093/nargab/lqae113

Neumann, P., Novák, P., Hoštáková, N. and Macas, J. (2019) Systematic survey of plant LTR-retrotransposons elucidates phylogenetic relationships of their polyprotein domains and provides a reference for element classification. Mobile DNA 10, 1. https://doi.org/10.1186/s13100-018-0144-1

Quinlan, A.R. and Hall I.M. (2010) BEDTools: a flexible suite of utilities for comparing genomic features. Bioinformatics 26, 841–842, https://doi.org/10.1093/bioinformatics/btq033

Smit, A.F.A, Hubley, R. and Green, P. (2013-2015) RepeatMasker Open-4.0. http://www.repeatmasker.org

Supplementary Dataset S1. Physical map of JI2822 showing precise location of deletions with respect to genes corresponding to phenotypic markers

The Figure below shows the chromosomal pseudomolecules of pea displayed in the relevant orientation, the position of the deletions in the quintuple vicilin mutant, and the location of genes corresponding to phenotypic markers. This shows the expected orientation of genetic markers on genetic maps, retaining the nomenclature used previously but reorienting the pseudomolecules in accordance with cytogenetical convention (see Background below). In addition, the locations of the 5S and 45S rRNA genes are indicated, as these are widely used for *in situ* hybridization studies of the pea karyotype. The locations of the extended centromeres in pea (Neumann et al. 2016) are indicated, with reference to regions of low recombination rate (Ellis et al. 2023).

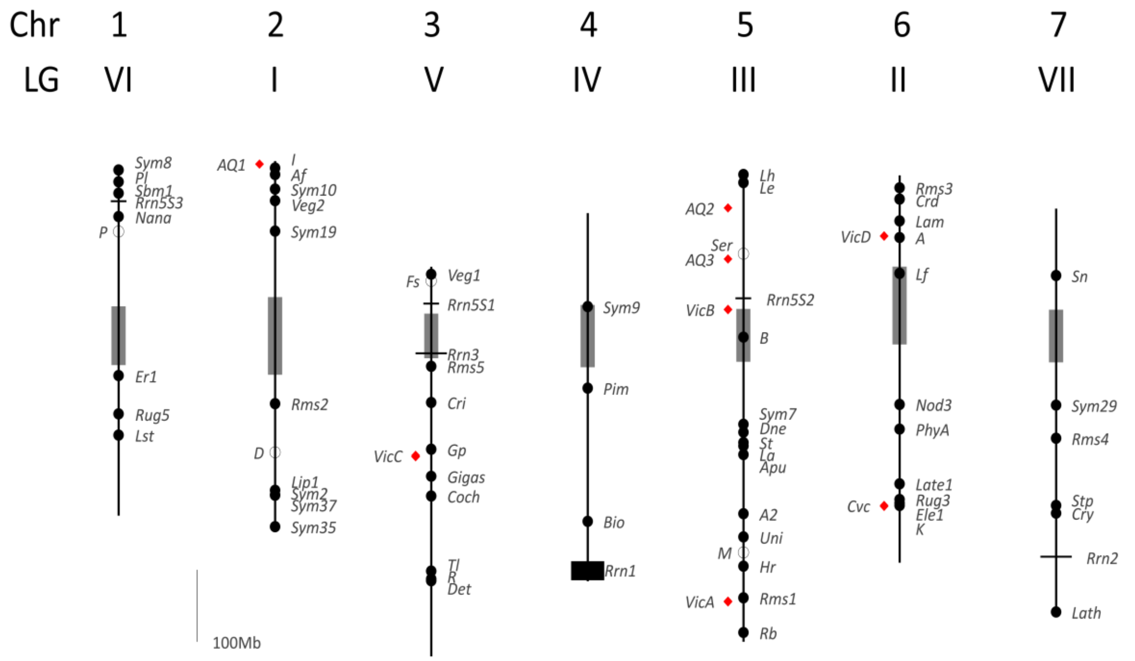

Figure legend: The position of the deletions in the quintuple vicilin mutant (red diamonds: *VicA-D, Cvc*, additional (AQ1-3)) and known genes on the JI2822 v1.2 assembly, as indicated on each pseudomolecule. The genes, with NCBI and JI2822 genome reference, where applicable, are: *A* (GU132942; PsatJI2822v1chr6G23680.1;), *A2* (HQ245307; PsatJI2822v1chr5G82300.1), *Af* (*PsPALM1b*; *PsPALM1a*), *Apu* (JQ653163), *B* (GU596478; PsatJI2822v1chr5G42400.1), *Bio* (MG515008; PsatJI2822v1chr4G65560.1), *Coch* (JN180864; PsatJI2822v1chr3G45020.1), *Crd* (HQ439907; PsatJI2822v1chr6G11680.1), *Cri* (AF299140; PsatJI2822v1chr3G21900.1), *Cry* (DQ845340; PsatJI2822v1chr7G65920.1), *Det* (AY340579; PsatJI2822v1chr3G61460.1), *Dne* (AY830926; PsatJI2822v1chr5G62140.1), *Ele1* (MG515010; PsatJI2822v1chr6G71620.3), *Er1* (FJ463618; PsatJI2822v1chr1G45780.1), *Gigas* (HQ538822; PsatJI2822v1chr3G39960.1), *Hr* (JN983406; JI2822 chr5:544925836-544929653), *I* (AB303331; PsatJI2822v1chr2G04280.1), *K* (EU574915; PsatJI2822v1chr6G72000.1), *La* (DQ848351; PsatJI2822v1chr5G64540.1), *Lam* (X88789; PsatJI2822v1chr6G20480.1), *Late1* (EF185297; PsatJI2822v1chr6G66420.1), *Lath*

(JQ291249; PsatJI2822v1chr7G91540.1), *Le* (U93210; PsatJI2822v1chr5G02880.1), *Lf* (AY343326; JI2822 chr6:135881325-135885412), *Lh* (AY245442; PsatJI2822v1chr5G00540.1), *Lip1* (AJ276591; PsatJI2822v1chr2G55080.1), *Lst* (EU574914; PsatJI2822v1chr1G57620.1), *Nana* (AF537321; PsatJI2822v1chr1G21720.1), *Nod3* (GU580938; PsatJI2822v1chr2G58440.1), *PhyA* (AY688953; PsatJI2822v1chr6G55120.1), *Pim* (AF461740; PsatJI2822v1chr4G37260.1), *Pl* (PsatJI2822v1chr1G07060.1), *R* (X80009; PsatJI2822v1chr3G60900.1), *Rb* (X96766; PsatJI2822v1chr5G109520.1), *Rms1* (AY557342; JI2822 chr5:588985623-588988744), *Rms2* (MG495397; JI2822 chr2:336274033-336276590), *Rms3* (KT321518; JI2822 chr6:17085398-17086467), *Rms4* (DQ403159; PsatJI2822v1chr7G45060.1), *Rms5* (DQ403160; PsatJI2822v1chr3G16040.1), *Rug3* (AJ250770; PsatJI2822v1chr6G70620.1), *Rug5* (X88790; PsatJI2822v1chr1G53180.1), *Sbm1* (AY423375; PsatJI2822v1chr1G12120.1), *Sn* (KJ801796; PsatJI2822v1chr7G21140.1), *St* (MF033127; PsatJI2822v1chr5G63780.1), *Stp* (AF004843; PsatJI2822v1chr7G64320.1), *Sym10* (AJ575252; PsatJI2822v1chr2G11940.1), *Sym19* (AF491997; PsatJI2822v1chr2G22300.1), *Sym2* (EU564087; PsatJI2822v1chr2G55660.1), *Sym29* (AJ495759; PsatJI2822v1chr7G36680.1), *Sym35* (AJ493064; PsatJI2822v1chr2G66140.1), *Sym37* (EU564102; PsatJI2822v1chr2G55640.1), *Sym7* (EU736106; PsatJI2822v1chr5G60000.1), *Sym8* (EF447280; PsatJI2822v1chr1G00680.1), *Sym9* (AJ621916; PsatJI2822v1chr4G25920.2), *Tl* (EU938525; PsatJI2822v1chr3G60120.1), *Uni* (AF010190; PsatJI2822v1chr5G88120.1), *Veg1* (JN974185; PsatJI2822v1chr3G02620.1), *Veg2* (KP739949; JI2822 chr2:54983850-54984812). Grey boxes delimit the extended centromeres located with respect to regions of extremely reduced recombination rates (Ellis et al. 2023). The ribosomal RNA genes are indicated: *Rrn1-3* for the 45S rRNA (nucleolus organizer regions) and *Rrn5S 1-3* for the 5S rRNAs.

The three expected sites for the 5S rRNA genes (Ellis et al. 1988, Simpson et al. 1990) are identified in the Figure. A fourth site of the 5S rRNA genes was identified in the pea accession Grüne Victoria (Fuchs et al. 1998), but this was not found in the JI2822 genome. The 45S rRNA gene arrays corresponding to *Rrn1* and *Rrn2* were located; these correspond to the nucleolus organizer regions of the secondary constrictions on chromosomes 4 and 7; however, the expected satellite for chromosome 4 is missing from the pseudomolecule. The extent of *Rrn2* appears to be smaller than would be expected, but there are extensive arrays of rRNA genes not incorporated within pseudomolecules, presumably because of the difficulty of assembling these homogeneous tandem repeats. Additional sites for the 45S rRNA genes were identified, the largest of which is indicated as *Rrn3*, but these additional 45S rRNA gene arrays do not correspond to known genetic or cytogenetic markers. It should be noted that the sites of rRNA genes are known to be highly variable in comparisons between closely related species, presumably reflecting the amplification and or contraction of arrays.

Background: By convention, karyotypes number chromosomes in descending order of size and orientate chromosomes such that short arms are uppermost. Early studies in pea genetics and cytogenetics sought to reconcile the numbering of chromosomes and linkage groups; to a large degree this was dependent on the segregation of classical genetic markers in crosses where translocations segregated. This effort unfortunately confounded several errors both in linkage analysis and in the assignment of translocations to individual chromosomes (Ellis and

Poyser 2002), and the conventional numbering of both chromosomes and linkage groups was lost. There were several different iterations of these numbering systems (Lamprecht 1948, Blixt 1959, Lamm and Miravalle 1959, Fuchs 1998, Neumann et al 2002, Tayeh et al 2015) which has led to confusion and the adoption of a compound chromosome plus linkage group numbering system for pea chromosomal pseudomolecules (Kreplak et al. 2019). Here we have not changed the nomenclature but have reorientated the pseudomolecules in accordance with cytogenetical convention.

References

Blixt, S. (1959) Cytology of *Pisum.* III. Investigation of five interchange lines and coordination of linkage groups with chromosomes. Agricultural and Horticultural Genetics: 17: 47-75.

Ellis, T.H.N. and Poyser, S.J. (2002) An integrated and comparative view of pea genetic and cytogenetic maps. New Phytologist 153: 17-25

Ellis T.H.N., Lee D., Thomas C.M., Simpson P.R., Cleary W.G., Newman M-A. and Burcham K.W.G. (1988) 5S rRNA genes in *Pisum*: Sequence, long range and chromosomal organization. Molecular and General Genetics 214: 333-342

Ellis, N., Hofer, J., Sizer-Coverdale, E., Lloyd, D., Aubert, G., Kreplak, J., Burstin, J., Cheema, J., Bal, M., Chen, Y., Deng, S., Wouters. R.H.M., Steuernagel, B., Chayut, N. and Domoney, C. (2023) Recombinant inbred lines derived from wide crosses in *Pisum*. Scientific Reports 13: 20408.

Fuchs, J., Kühne, M. and Schubert, I. (1998) Assignment of linkage groups to pea chromosomes after karyotyping and gene mapping by fluorescent *in situ* hybridization. Chromosoma 107: 272-276

Kreplak J, Madoui M-A, Cápal P, Novák P, Labadie K, Aubert G, Bayer PE, Gali KK, Syme RA, Main D, Klein A, Bérard A, Vrbová I, Fournier C, d'Agata L, Belser C, Berrabah W, Toegelová H, Milec Z, Vrána J, Lee HT, Kougbeadjo A, Térézol M, Huneau C, Turo CJ, Nacer Mohellibi N, Neumann P, Falque M, Gallardo K, McGee R, Tar'an B, Bendahmane A, Aury J-M, Batley J, Le Paslier M-C, Ellis N, Warkentin TD, Coyne CJ, Salse J, Edwards D, Lichtenzveig J, Macas J, Doležel J, Wincker P, Burstin J. (2019) A reference genome for pea provides insight into legume genome evolution. Nature Genetics 51: 1411–1422.

Lamm, R. and Miravalle, R. J. (1959) A translocation tester set in *Pisum*. Hereditas 45: 417-440.

Lamprecht, H. (1948) Further studies of the linkage group *Cp—Gp—Fs—Ast* of *Pisum sativum*. Agricultural and Horticultural Genetics 6: 1–9

Neumann, P., Pozárková, D., Vrána, J., Dolezel, J. and Macas, J. (2002) Chromosome sorting and PCR-based physical mapping in pea (*Pisum sativum* L.). Chromosome Research 10: 63-71.

Neumann, P., Schubert, V., Fuková, I., Manning, J.E., Houben, A. and Macas, J. (2016) Epigenetic Histone Marks of Extended Meta-Polycentric Centromeres of *Lathyrus* and *Pisum* Chromosomes. Frontiers in Plant Science 7:234. doi: 10.3389/fpls.2016.00234

Shirasawa, K., Sasaki, K., Hirakawa, H. and Isobe, S. (2021) Genomic region associated with pod color variation in pea (*Pisum sativum*), G3 Genes|Genomes|Genetics, 11: jkab081

Simpson P.R., Newman M-A., Davies D.R., Ellis T.H.N., Matthews P. and Lee D. (1990) Identification of translocations in pea by *in situ* hybridization with chromosome specific DNA probes. Genome 33: 745-722

Tayeh, N., Aluome, C., Falque, M., Jacquin, F., Klein, A., Chauveau, A., Bérard, A., Houtin, H., Rond. C., Kreplak, J., Boucherot, K., Martin, C., Baranger, A., Pilet-Nayel, M.-L., Warkentin, T.D., Brunel, D., Marget P., Le Paslier, M.-C., Aubert, G. and Burstin, J. (2015) Development of two major resources for pea genomics: the GenoPea 13.2K SNP Array and a high density, high resolution consensus genetic map. The Plant Journal 84: 1257–1273

Zhang, P.P., Wang, Y.G., Sun, T., Wu, X.Y., Xia, W.J., Fang, P.P., Pandey, A.K. and Xu, P. (2022) Fine mapping *PsPS1*, a gene controlling pod softness that defines market type in pea (*Pisum sativum*). Plant Breeding **141**: 418-428.
